# Systematic assessment of antibody selectivity in plasma based on a resource of enrichment profiles

**DOI:** 10.1038/s41598-019-43552-5

**Published:** 2019-06-06

**Authors:** Claudia Fredolini, Sanna Byström, Laura Sanchez-Rivera, Marina Ioannou, Davide Tamburro, Fredrik Pontén, Rui M. Branca, Peter Nilsson, Janne Lehtiö, Jochen M. Schwenk

**Affiliations:** 1 Division of Affinity Proteomics, Science for Life Laboratory, Department of Protein Science, KTH - Royal Institute of Technology, 171 21 Solna, Sweden; 2 Cancer Proteomics, Department of Oncology-Pathology, Science for Life Laboratory, Karolinska Institute, 171 21 Solna, Sweden; 3 Department of Immunology, Genetics and Pathology, Science for Life Laboratory, Rudbeck Laboratory, Uppsala University, 751 85 Uppsala, Sweden

**Keywords:** Blood proteins, Immunoprecipitation

## Abstract

There is a strong need for procedures that enable context and application dependent validation of antibodies. Here, we applied a magnetic bead assisted workflow and immunoprecipitation mass spectrometry (IP-MS/MS) to assess antibody selectivity for the detection of proteins in human plasma. A resource was built on 414 IP experiments using 157 antibodies (targeting 120 unique proteins) in assays with heat-treated or untreated EDTA plasma. For each protein we determined their antibody related degrees of enrichment using z-scores and their frequencies of identification across all IP assays. Out of 1,313 unique endogenous proteins, 426 proteins (33%) were detected in >20% of IPs, and these background components were mainly comprised of proteins from the complement system. For 45% (70/157) of the tested antibodies, the expected target proteins were enriched (z-score ≥ 3). Among these 70 antibodies, 59 (84%) co-enriched other proteins beside the intended target and mainly due to sequence homology or protein abundance. We also detected protein interactions in plasma, and for IGFBP2 confirmed these using several antibodies and sandwich immunoassays. The protein enrichment data with plasma provide a very useful and yet lacking resource for the assessment of antibody selectivity. Our insights will contribute to a more informed use of affinity reagents for plasma proteomics assays.

## Introduction

Antibodies are important tools used in a wide range of assays within life science, but there is a growing awareness about the importance to carefully validate the data generated^1^. To address this challenge, the recently formed International Working Group for Antibody Validation (IWGAV) proposed five strategies to assess the experimental performance of antibodies^2^. However, there is a need to expand the analytical possibilities for evaluating antibodies in the given context (=  sample type) and application (= assays). In particular for body fluids there are, besides adding or depleting a protein of interest, currently no tools for modulating the system to overexpress or inhibit expression of a target of interest. Performing correlation analysis using paired antibodies or orthogonal methods is a commonly used approach. Recently, the use of correlation between the variation found in loci of the genome and plasma protein profiles provides a powerful method to determine if the information in a gene of interest or encoded elsewhere in the genome is driving differences in plasma abundance^3,4^. Such studies allow to infer the specificity of an affinity reagent, but they do not provide direct and molecular information about the physical interaction between the protein target and affinity reagent. Hence, there is still a missing element to experimentally assess selectivity of the affinity reagents and to enable the development of reliable and sensitive assays for protein analysis in fluids such as plasma or serum.

Here, we describe our efforts and observations from the assessment of antibody selectivity in plasma. We utilized immunoprecipitation (IP) of endogenous proteins in combination with shotgun mass spectrometry (MS) to systematically analyze the proteins enriched by the antibodies. Similar strategies have been applied previously on cellular level to identify interactors and co-immuno-precipitated targets in protein-protein interaction studies^5,6^. Until now, the utility of shotgun MS for a systematic antibody validation has been less explored, however, Marcon and colleagues evaluated the performances of 1,000 recombinant antibodies for IP in cell lysates^7^. A more recent study presented an approach to generate and validate even 1,400 antibodies for IP of transcription factors^8^. While apart from studies using cell lysates and those focused on specific, smaller number of targets^9,10^, there are no systematic studies applying IP of endogenous full-length proteins and MS for antibody validation in human plasma. For plasma, trypsin digestion and peptide enrichment has been more frequently applied in combination with MS readout for quantification^11–13^. Additional approaches such as iMALDI^14^ and MS-based immunoassays^15^ complement activities using protein-enrichment before MS analysis in plasma.

The composition of the plasma proteome was recently updated and now lists around 3,500 proteins detectable using MS techniques^16^. It is well-known that only a small set of about 20 abundant proteins make up ~90% of the total protein content, hence the protein content and distribution of protein concentrations differs greatly when compared to cellular samples. For MS-based techniques, one of the keys is to use enrichment as a strategy to enhance the sensitivity for the quantification of a peptide/protein of interest^17^. Purely affinity-based techniques will greatly benefit from the utility of highly selective binder in multiplexed assay systems^18^. Understanding how other plasma proteins, such as the abundant and frequently observed contaminants, contribute to enrichment profiles will consequently improve the utility of affinity reagents for plasma proteomics assays.

We performed and compared the label free quantification (LFQ) MS data from more than 400 IPs, to build a library with the detected proteins and annotated them using frequencies of identification (ƒ) and z-scores (z). This assisted us in identifying those proteins detected with high frequency as “plasma background contaminants”. As a novelty in respect to previous studies focused on immune-precipitation^6,7^ we wanted to overcome the problem of background contaminants by considering the large number of independent IPs produced as a population. Previous studies compared replicated IPs for a specific antibody against IPs performed with same IgG species as negative control using fold-changes, univariate statistics and p-values as assessment criteria. We found z-scores a convenient statistical approach for the scope of our study, as they determine whether a specific sample represented or deviated from the populations of samples population or if it deviates. This approach allowed us to identify the endogenous proteins that were either most uniquely or commonly enriched by each antibody in the analyzed plasma sample. Statistics using z-scores have been widely used in clinical population studies^19^ and for the analysis of omics data types^20–22^ but not in the contest of the analysis of immunoprecipitation data. These scores built the foundation of our resource that we used for the systematic evaluation of antibodies selectivity in plasma by IP-MS/MS, where data analysis is often complicated by the high number of proteins commonly identified in each experiment.

## Results and Discussion

### Study overview

**Figure 1:**
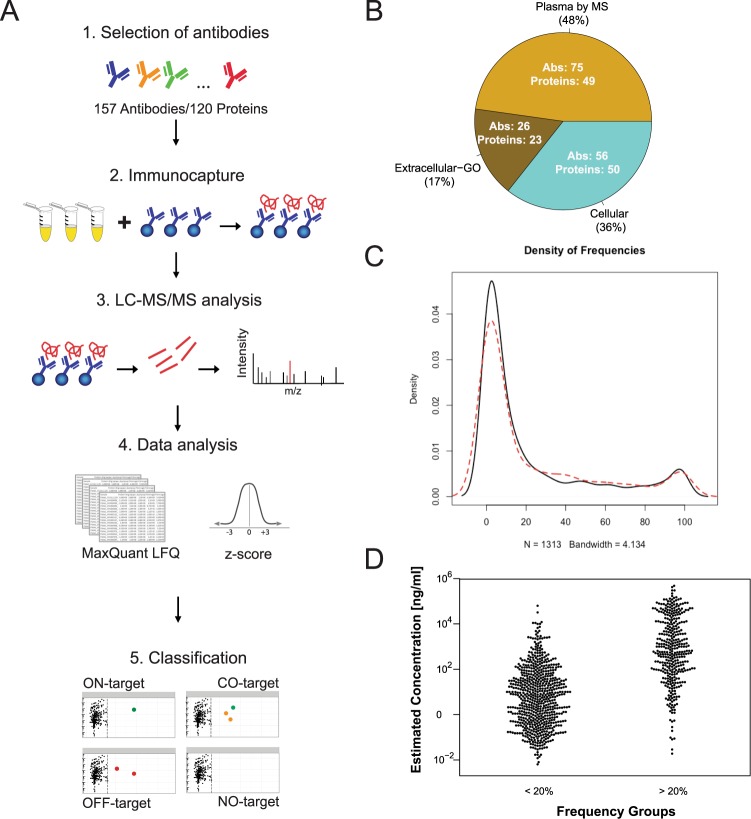
(**A**) Workflow and study overview. Antibodies were covalently coupled to magnetic beads and incubated one-by-one with EDTA plasma, and between 2–4 replicated incubations were performed for each antibody. Following target enrichment, washing and digestion on beads, the obtained data files from LC-MS were searched and normalized by MaxLFQ. Then z-score analysis was performed to rank proteins specifically enriched by each antibody. Using the resource generated by >400 IP assays, antibodies were classified based on their enrichment profiles: (1) ON-target, only the target protein was enriched showing a z-score ≥ 3 (2) CO- target, the target protein was enriched together with other proteins also associated to a z-score ≥ 3 (3) OFF- target, only proteins other than the expected target were enriched; as well as (4) NO- target, in case no protein was enriched (z-scores < 3). (**B**) Distribution of antigen annotation. The target proteins of the 157 antibodies were grouped as follows: “Plasma by MS”, were identified in plasma previously by mass spectrometry as reported by Peptide Atlas. Cellular and Extracellular were assigned according to Gene Ontology classification (see Materials and Methods). Numbers stated inside the pie chart refer to the number of antibodies (Abs) in the category and corresponding number of target proteins. (**C**) Distribution of frequencies of identification. The 1313 proteins obtained from the IP-MS/MS assays conducted in heat-treated (red) vs untreated plasma (black) were collected in terms of the number of times they were observed in the IP-MS data. For both sample types, the majority of the 1313 proteins were found in less than 20% of the IPs. (**D**) Frequency vs Concentration. Estimated concentrations reported in PeptideAtlas were compared between frequent (>20%) and less frequent (<20%) protein identifications.

To establish a plasma-centric resource for selectivity analysis of antibodies, we applied a common workflow to analyze 157 antibodies (targeting 120 proteins) (Fig. 1A). The assays were built on a previously described procedure, in which antibodies are covalently coupled onto magnetic polystyrene beads prior to incubation with the sample^23^. Following target enrichment, washing and digestion on beads, the data files obtained from LC-MS were searched and normalized by MaxLFQ. Next, z-score analysis was performed to rank proteins specifically enriched by each antibody. The study included mostly polyclonal binders from the Human Protein Atlas (HPA) but also monoclonal antibodies from mouse and other species. In order to compare the performance of different antibodies raised against a common antigen, a subset of 25 proteins (21%) were targeted by more than one antibody (S-Fig. 1A). The selection of presented targets was driven by giving priority to antibodies raised against proteins known to be part of the plasma proteome and to those associated to a disease (S-Excel Table, sheet: “Selected Targets”). As described in Fig. 1B, the majority of binders (65%, N = 101) were raised against target proteins detected previously ‘in plasma’ (48% N = 75) or annotated as ‘extracellular’ (17%, N = 26), and fewer proteins were annotated as ‘cellular’ (36%, N = 56). As reference for protein abundance in plasma, we considered the estimated concentrations found in the 2017 version of the plasma proteome draft hosted in PeptideAtlas^16^. In order to reduce the number of MS runs and reagent consumption, at least two replicated incubations per antibody were performed. The established resource was comprised of >400 IP assays, hence it was possible to compare and classify the antibodies based on whether their enrichment profiles showed the expected target proteins or not.

### Plasma samples

The measurement and data obtained in this study have been acquired in compliance with the Declaration of Helsinki for research on humans. The research was conducted on pools of anonymous donors and did not require sensitive personal information about the donors. The research did not include any type of intervention, surgery, or treatment. The Ethical Review Board in Uppsala (Dnr 2009/019) deemed that this research was not subjected to formal ethical review and approval. For the first 139 IPs, we used a pool K2 EDTA plasma samples collected by the Department of Laboratory Medicine (LABMED) at Karolinska hospital under a protocol approved by the Ethical Review Board in Stockholm (Dnr 2015/1570-31/4) and with written informed consent was obtained from all individuals. For the remaining 275 IPs, samples of human K2 EDTA plasma were purchased on two occasions from Sera Laboratories International Ltd (HMPLEDTA2, now part of BioIVT, West Sussex, UK), who collects samples under IRB-approved protocols in use at their FDA-licensed donor centers with written informed consent was obtained from all donors. The pools of plasma samples that were generated by the supplier from mixing plasma from donors of which 50% were females.

### Assessing the resource

It is well accepted that MS provides in-depth information about the protein content of a sample, however hundreds if not thousands of proteins can be identified in a single IP experiment^24^. This calls for a careful assessment and interpretation of data from IP assays where many other proteins than the intended target can be identified in the same range of spectral counts or precursor intensities. As described in the context of cell lysates, the necessity to compare the outcome of several experiments, including negative controls or unrelated antibodies is essential^5–7^. Mellacheruvu and colleagues showed how lists of background proteins that were obtained from negative control assays (which were performed in similar experimental conditions), and that even different experiments from different laboratories can be used to assess specific enrichments in IPs^5^. In our case, we aimed to build and use a large data set to compare all the IPs and not only those of control measurements with IgG matching the species of the primary antibody. We generated data in separate experiments, referred to as batches, in order to analyze our data. Building a large library of IP data, we wanted to extract information about background contaminants in plasma and annotate all identified proteins by their frequency of identification (ƒ) and antibody-related enrichment z-scores (z). This collection of data files could then serve as a resource to assist the interpretation of IP-MS/MS data when used for the assessment of antibody selectivity in plasma.

The data containing a total of 414 plasma IPs were prepared in 6 independent experimental batches (S-Excel Sheets “Experimental Batches”), and the raw data was analyzed in one unique session of MaxQuant. Applying the function MaxLFQ for label free quantification, a total of 1,313 unique proteins were identified, excluding components such as the variable domains of immunoglobulin for heavy and light chains. The resulting list of proteins can be found in S-Excel Sheet: “Frequencies of identification”. To provide an overview of the data, we performed two ways hierarchical clustering analysis and principal component analysis (S-Fig. 3A,B), which showed that IPs prepared during the same experimental batch and sample treatment type clustered together. As discussed previously by Mellacheruvu, small variation in sample-to-sample preparation may indeed influence protein recovery and therefore the number of identifications^5^. Nevertheless, the comparison of IP data from different experimental batches will allow us to determine true interactors over background contaminant given that we used similar experimental settings. In our study, a major difference between IPs experiments was the use of different batches of plasma and whether plasma was heat-treated or not (S-Fig. 3A). Samples experiencing heat treatment clustered together independently from the experimental batch. As further discussed below, an evident difference in the proteins commonly recovered in the IP procedure included mostly background contaminants.

In Fig. 1C, the distribution of frequencies of protein identifications is shown. The proteins obtained from assays conducted in heat-treated (red) vs untreated plasma (black) were collected in terms of the number of times they were observed in the IP-MS data. Comparing the proteins detected for each of the pre-treatment types, the majority (66%) of the 1313 proteins were found in less than 20% of the IPs. In Fig. 1D, we connect these frequencies with the estimated concentrations as provided by the 2017 draft of the plasma proteome hosted by PeptideAtlas^16^. We called those proteins frequent if they appeared in >20% of the IPs and less frequent if there were identified in <20%. Protein LFQ intensity values showed poor correlation with estimated concentration from PeptideAtlas and z-scores (S-Fig. 4C,D). Protein composition of IP samples was expected to be different from crude plasma, even if the proteins most abundant in plasma tend to be also the most abundant contaminants. The z-score values, which indicate the proteins mostly enriched in a specific IP, were not necessarily related to the abundance of the protein in plasma. The z-scores also did not correlate with LFQ intensities (S-Fig. 4E). LFQ intensity provides semi-quantitative values of abundance and very high LFQ values were observed for high abundant background contaminants. LFQ values did not directly indicate the most enriched proteins.

The resulting GO analysis revealed that terms related to the complement activation and wound healing (GO:0002576, GO:0006956, GO:0050817, GO:0009611, GO:0007596) were enriched for the frequent proteins (S-Excel Table Sheet: “GO enrichment analysis”). Other terms enriched for the frequent proteins were related to lipoprotein and their complexes (GO:1990777, GO:0032994, GO:0034358) as well as vesicles (GO:0031983, GO:0060205). Further details on frequency of identification and intensities along with insights on the impact of experimental batches can be found in the S-Excel Table Sheet:”Batches Kruskal Wallis test”.

### Effect of heat treatment

Previously, we have shown that heat treatment of plasma samples at 56 °C, which is also often used in proteomics sample preparation to enhance reduction and alkylation, can improve the limit of detection of some proteins in plasma profiling assays using antibody bead arrays^25,26^. Heat treatment has also been shown to improve the detection of proteins from the complement system^27^. Depending on the proteins, heat treatment of plasma may indeed have diverse effects on their analytical detectability. Consequences of heat-induced unfolding could lead to protein complexes breaking up and retrieve previously hidden epitopes. This would facilitate proteins to be more accessible to antibody binding. However, heat may also cause the proteins to aggregate and precipitate^28,29^ or to become more prone to non-specific binding^30^. These effects could either lead to a loss in protein of interest or be beneficial if the amount of off-target proteins can be reduced.

**Figure 2:**
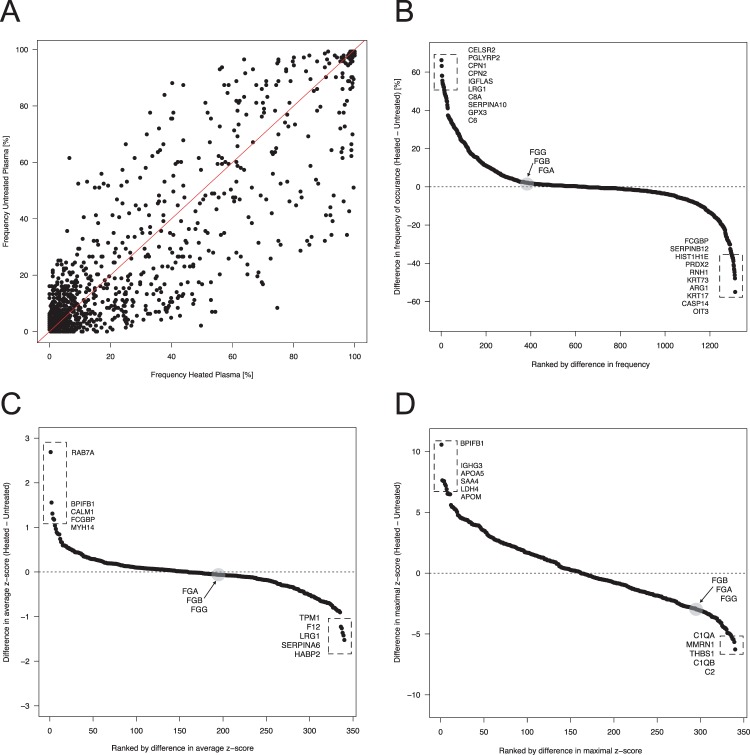
Comparison of heat-treated and untreated plasma. (**A**) The relation between the frequencies of all identified proteins in heat-treated and untreated plasma is shown. The red line represents the line of identity. The Spearman correlation was rho = 0.83 (p < 2 × 10^**−16**^). (**B**) Using the differences between frequencies in heat-treated and untreated samples highlights the proteins prone to be more commonly detected in either preparation. This is related to the fibrinogens (FGA, FGB, FGG), which were the proteins found to be most different when using LFQ intensity as a measure of abundance (S-Fig. 5A–C). In (**C**) is the comparison of the enrichment using the difference in average z-scores between the 340 proteins noted as common contaminants in both heat-treated and untreated plasma. (**D**) Comparison of enrichment scores of using the difference in maximal z-scores between the 340 proteins.

As shown for all IPs in Fig. 2A,B, we compared the frequencies of protein identification in heat-treated and untreated samples. We defined those proteins that were detected in either sample in >20% of all assays as contaminants. We found a total of 444 proteins in heat-treated and 389 proteins in untreated plasma. There were 104 proteins classified as contaminants only for heat-treated plasma and 49 proteins classified as contaminants only for untreated plasma. The fusion of these lists were 493 proteins, of which 340 (69%) were detected in >20% of the assays in both types of sample preparations. In addition to the frequency, we also compared the average and the maximal z-scores determined for the 340 common contaminants (Fig. 2C,D). Highlighted are the most differential proteins in terms of the z-scores and we related these to fibrinogens (FGA, FGB and FGG), as discussed below.

Further investigations also found a significant association (p-value < 2 * 10^−16^) between the frequencies and estimated protein abundance in plasma (Fig. 1D and S-Fig. 4A,B). When we considered LFQ intensities, we observed that in heat-treated samples particularly fibrinogens (FGA, FGB and FGG) were more abundant (S-Fig. 5A,B). All three fibrinogen chains were otherwise detected in >98% of all samples. Their maximum z-scores were z < 1.8 in untreated plasma compared to z > 4.4 in heat-treated plasma. Fibrinogen was enriched particularly in the IPs performed in not-heated plasma with anti-LCN2 antibodies and anti-Furin antibodies (S-Fig. 5C and Supplementary Excel Table, Sheet z-scores > 2.5). Previous observations state that fibrinogen is particularly affected by heat-treatment at temperature close to 56 °C. Denaturation of fibrinogen starts at 55 °C and this property was exploited in the past to develop fibrinogen assays^28^. At this temperature the D fragment is particularly affected^29^. The affinity of fibrinogens to plastic surfaces has also been reported to increase in heat-treated samples^30^. Via a similar mechanism, fibrinogen’s unspecific binding to surfaces of magnetic beads or to heavy chains of IgG antibodies coupled onto the beads may be enhanced^31,32^. Hence, heat-induced denaturation of fibrinogen could have altered the magnitude with which proteins of this family are identified via a variety of mechanisms.

Another example is given for fibulin 1 (FBLN1), a frequent and abundant plasma protein (ƒ = 65%; [c] = 34 µg/ml). A monoclonal antibody raised against FBLN1^33^ enriched a total of 22 proteins in heat-treated and 12 in untreated plasma. FBLN1, however, was only among these enriched proteins in heat-treated plasma (z = 3.7). This indicated that heat-treated plasma was the preferred condition of this antibody to enrich this abundant target protein. Other plasma proteins such as albumin, apolipoproteins (APOB, APOC2, APO2, APOE), Keratines (KRT1, KRT2, KRT10), Fibulin, Fibronectin 1 or IgM were less common among the frequent contaminants (see also S-Excel Sheet: “Frequencies of identification”).

### Analysis of selectivity

In the following, we describe our approach to investigate antibody selectivity in plasma. Knowing that proteins commonly identified as background in plasma may differ between assays due to how plasma was treated before the experiment, we calculated the z-scores from the respective assays using heat-treated and untreated plasma separately (S-Fig. 10). We found that the average number of identifications per experiment were slightly higher in heat-treated plasma (300 ± 97 in 276 IPs) compared to untreated plasma (283 ± 97 in 138 IPs). We considered a protein as being enriched by an antibody in EDTA plasma, which was derived from pool of healthy donors, when the z-score ≥ 3. For the antibodies used to build this resource, we found a total of 600 unique proteins above this threshold.

**Figure 3:**
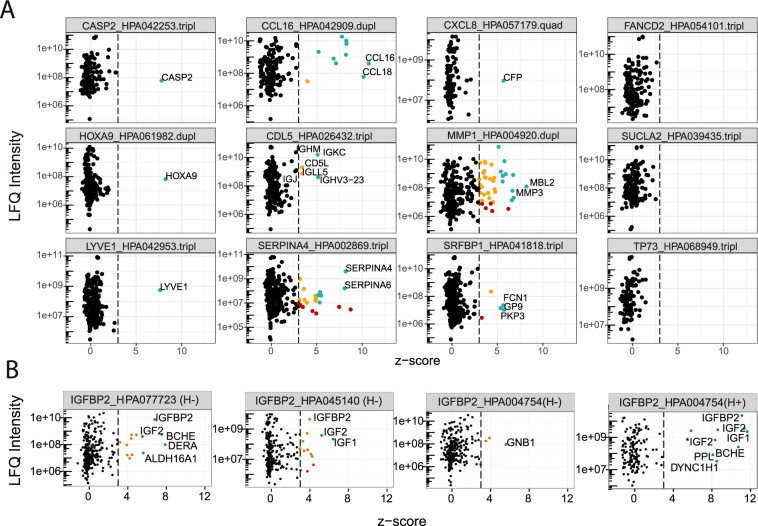
(**A**) Classification of antibodies. Three representative examples are shown for each of the enrichment categories (ON-target, CO-target, OFF-target, and NO-target). On the top of each plot are the target gene, antibody ID and the number of replicated IP performed for the antibody. The dots in each plot represent protein identifications made in all the replicates available for the specific antibody. Green dots: z-score >5 and LFQ intensity >1e + 07; yellow dots: z-score > 3 and LFQ intensity >1e + 07; red dots: z-score > 3 and LFQ intensity <1e + 07. Text: expected target and hypothesized off-targets or interactors. A complete list of identified protein and relative z-scores are available in Supplementary Excel Table (Sheet: z-score > 2.5). (**B**) Paired antibodies and co-enrichment profiles. The z-score/LFQ intensity plots of paired antibodies raised against IGFBP2 are shown for HPA004754, HPA045140, HPA077723. (H+) refers to heat treated plasma and (H−) to untreated plasma. IGF2 was identified as P01344, and P01344-2 (*).

All antibodies were annotated according to the following categories (Fig. 3A):

(i) **Supportive**: **The expected target was enriched with a z-score ≥ 3 was assigned**.

- **ON-target category**: when a z ≥ 3 was only assigned to the expected target.
- **CO-target category**: when a z ≥ 3 was assigned to the expected target but also other proteins besides the expected targets were detected with z ≥ 3.

(ii) **Uncertain: The expected target was not identified or enriched with z-score < 3**.

- **OFF-target:** other proteins than the expected targets were enriched with z ≥ 3.
- **NO-target:** all detected proteins were classified with z < 3.

The outcome of the analyses is also shown in Tables 1 and 2, where the classification of 70 out of 157 antibodies (45%) was denoted as ‘supportive’. When assessing only those antibodies targeting proteins previously annotated in plasma, the fraction increased to 61% (46/75). We noted that in almost all of the supportive evaluations, the highest z-score and the highest LFQ intensity was assigned to the expected target, and therefore this protein can be considered as the primary target even though other proteins were identified.

**Table 1:** Annotation of antibodies.

Annotated target location	Supportive		Uncertain		Sum	
	N	%	N	%	N	%
Cellular	15	27	41	73	56	36
Extracellular	9	34	17	65	26	16
Plasma (MS)	46	61	29	39	75	48
Total	70	45	87	55	157*	100

*Total number of antibodies used in the study, including 4 antibodies used only in the phase of optimization, for which a z-score was not calculated. These 4 antibodies were anyway classified as supportive if they immuno-precipitate and enriched their target.

**Table 2:** Annotation and categorization and of antibodies by subcategories.

	N	%
On-target	11	7
Co-target	57	37
Off-Target	72	47
No-Target	13	9
Total	153**	100

**Antibodies classified in the four sub-categories according to z-scores.

We defined the category of IPs where we could not detect the intended target as ‘uncertain’, and we acknowledge that there are several different factors leading to this observation: It could be that the actual target is (i) not present in the sample derived from pooling those from healthy donors, (ii) not accessible due to aggregation or formation of complex or (iii) present but at a concentration below the limit of detection of our method. Additionally, the presence of an extensively higher abundant off-target could either mask the binding site or limit the detectability of target peptides. A limited performance, such as ionization, could also be intrinsic to the peptides of the target itself.

### ON-target enrichment

Applying IP-MS analysis has been reported to improve the sensitivity of protein quantification^11,15,34^. Hence, IP-MS may allow the detection of lower abundant proteins, aiding to identify those proteins that presently remain more challenging for other MS protocols. Our investigations lead to the identification of 9 extracellular proteins (e.g. CXCL8, TGFA) and 15 cellular proteins (e.g. TP53, CASP2) in plasma, that were not listed in the plasma PeptideAtlas at the time of our study Sheet: “Antibodies experim. annotation”. For almost 50% of the antibodies annotated as ON- or CO-target, the identified peptides aligned with the sequence of the antigens used to generate and affinity purify the antibodies (S-Fig. 1B). As previously discussed^35^, affinity purification of antibodies may bare the risk of co-eluting the target used as bait from the columns and thereby carry the baits over into the assay. These ‘passenger’ proteins or peptides may consequently simulate the enrichment of an endogenous target. In our case, this would lead to a false positive classification of the antibody and may hamper the performance of downstream applications. To address this concern, we analyzed antibody-coupled beads for the presence of the protein fragment that was used as antigen and that could have introduced the passenger peptides. Out of 47 tested antibodies, 11 indicated a possible presence of passenger proteins (S-Fig. 6), from which we categorized 1 as ON-target, 1 as OFF-target and 9 as CO-target.

### CO-target enrichment and sub-categories

Evaluating antibodies in terms of target selectivity (Fig. 3), the CO-target category includes those antibodies for which other proteins were enriched alongside the intended target. In our study, most antibodies were categories in this group, suggesting that single-binder assays frequently detect more proteins than only the intended targets. The reasons for observing additional proteins could be due to (i) off-target binding by the antibodies (direct co-enrichment) or due to (ii) an interaction of the intended target with another protein (indirect co-enrichment).

For the first sub-category, sequence homology and abundance of the OFF-target could serve as reasons for co-enrichments. In the coming section, we focused on using our frequency and z-score values to annotate the co-targets and relate these to the plasma concentration values estimated by PeptideAtlas. While it remains necessary to investigate at which concentration ratios on- or off-target binding is dominating, using existing data and sequence homology searches can provide a first lead for judging the selectivity of an antibody. For the second sub-category, we propose to use paired antibodies against a common target in order to determine indirect co-enrichment. As presented further below for the example IGFBP2, we shown how our IP data can be used to identify interacting proteins in plasma and guide the development of immunoassays for these interacting proteins.

### CO-target enrichment stemming from related proteins

Examples of CO-target enrichment driven by sequence homology are presented for antibodies raised against CCL16 (HPA042909) and SERPINA4 (HPA002869). Both of these binders enriched additional members of the respective protein families, namely CCL18 and SERPINA6, which both shared sequence homology with the intended on-target.

The proteins CCL16 and CCL18 are estimated to be present at 29 ng/ml and 2 ng/ml levels in human plasma. The estimated 15-fold difference in abundance was not found when comparing the z-scores to which HPA042909 captured CCL16 (z = 10.7) and CCL18 (z = 10.1). Both proteins were otherwise rarely observed in any of the other IPs (ƒ < 4%) and they have not been predicted to interact directly with another (S-Fig. 7A), however they do share a 27% sequence homology (S-Fig. 7B). In the case of HPA002869, the antibody enriched SERPINA4 (z = 8.2) and SERPINA6 (z = 8.1) in heat-treated plasma. SERPINA4 and A6 are estimated to be present at 17 and 41 µg/ml levels (2.4-fold difference) and share a 40% sequence similarity (S-Fig. 7D). Both proteins were observed in 91% (SERPINA4) and 59% (SERPINA6) of all conducted IPs with heat-treated plasma and found to be less frequent in untreated plasma. Serpins are a large family of highly homolog blood proteins but no direct interactions between the members have yet been observed in the String database (S-Fig. 7C). However, information found in another database of predicted human protein-protein interactions^36^ (http://www.compbio.dundee.ac.uk/www-pips/index.jsp) indicated an interaction between SERPINA4 and SERPINA6 (total interaction score = 128.0). Further experiments will be needed to determine if SERPINA4 and A6 indeed interact or if HPA002869 shares specificity for both of these proteins.

Approaches to differentiate between direct or indirect co-enrichment could, for example, include to spike in the off-target in presence and absence of the on-target (see analysis of IGFBP2 interactions further below). Otherwise, diluting the samples that contain both on- and off-targets at roughly equal concentrations can be used to determine if the ratio between the detected amounts of on- or off-target changes at a certain sample dilution. Optimally, several antibodies with independent epitopes would be needed to investigate the possibility that interactions between these proteins exist. Since we did not find literature supporting the actual physical interaction between the proteins discussed in the examples above, we would judge the described antibodies still as valuable for the development of sandwich immunoassays, because the specificity of a second antibody will add certainty about which protein is being measured.

### CO-target enrichment of frequently observed and abundant proteins

In another sub-category, protein enrichment may also be driven by more frequently observed and abundant proteins, for which z-scores tend to remain <3 in our resource.

An example is given by CD5 antigen-like (CD5L, [c] = 5.9 µg/ml), which was detected in almost all IPs together with IGHM (ƒ > 99%). It is indeed known that CD5L binds to the Fc region of IgM through its SRCR domains^37^. Further to this, the immunoglobulin J chain (IGJ) is known to be required to stabilize the binding of CD5L to IGM, but a direct interaction has not been experimentally observed^38^. Using HPA026432 to enrich CD5L (z = 5.1), we also detected immunoglobulin light chain lambda (IGHV3-23; z = 3.4), kappa (IGKC; z = 3.4), IGM (z = 2.7) and IGJ (z = 2.8). The distribution of z-scores among these known binding partners may also possibly indicate that the antibody is more selective for CD5L rather than for the additionally identified proteins. Considering the abundance of IGM at around 1 mg/ml and that IGM frequently appeared as contaminant (ƒ = 98%), an increased z-score for IGM in this particular IP pointed at a more specific enrichment due to the interaction with the primary target CD5L.

In order to determine how and if more frequent and abundant proteins interact, become off-targets or interfere with detection of the intended target, it is suggested to further dilute the plasma samples. Using the currently applied assay conditions (1:10 plasma dilution), it remains a challenge to judge the enrichment profiles of antibodies raised against the more frequently observed proteins. Further analyses will be needed to determine the mode of co-enrichment, meaning, if the co-target was detected due to interacting with the on-target or due to be serving as an off-target for the antibody.

### Studying protein interaction with paired antibodies

A particularly interesting annotation category grouped those antibodies for which physiologically meaningful interactions between the intended target and the additional proteins could be expected. To limit the search space, we chose stringent criteria to z ≥ 5 and LFQ intensity ≥10^7^ before calling a protein a potential interactor. In general, we observed that most of the consistent identifications (identified in several replicates) were found for LFQ precursor intensities above this level (S-Fig. 8D). In addition, examples of potential protein interactions should preferably be limited to those proteins for which multiple antibodies raised against different antigens of same target protein revealed matching protein interaction profiles. In such cases, concordant enrichment data of both ON-target and CO-targets provides supportive evidence for protein complexes rather than artefacts. The example we chose to highlight from our study was the insulin growth factor binding protein 2 (IGFBP2), but other examples existed for FBLN1 and IGF1R (see S-Excel Table Sheet: “Antibodies against same protein”).

The example given here used three antibodies raised against IGFBP2 (HPA077723, HPA045140, HPA004754) of which the latter two were raised against the same antigen. The binders revealed consistent identifications for LFQ intensities >10^7^ (S-Fig. 8E–H). As shown in Fig. 3B for untreated plasma, HPA077723 and HPA045140 both enriched IGFBP2 ([c] = 1.1 µg/ml; ƒ = 21%) as well as previously known interactors insulin growth factor 1 (IGF1: [c] = 0.46 µg/ml; ƒ = 18%) and IGF2 ([c] = 1.6 µg/ml; ƒ = 8%). In addition, the proteins butyrylcholine esterase (BCHE: [c] = 11.0 µg/ml; ƒ = 18%) and deoxyribose-phosphate aldolase (DERA: [c] = 0.5 ng/ml; ƒ = 7%) were detected with HPA077723. For the third binder HPA004754, IFGBP2 and BCHE were only enriched upon prior heat treatment of plasma (Fig. 3B), even though the antibody was raised against the same antigen as HPA045140. This difference in performance indicated the necessity to investigate each of the different batches and lots separately when using polyclonal antibodies. While an interaction and functional relationship between BCHE and IGF1 was previously hypothesized^39,40^, the concordant enrichment data from two different antibodies suggests that both proteins may likely be bound another in plasma.

**Figure 4:**
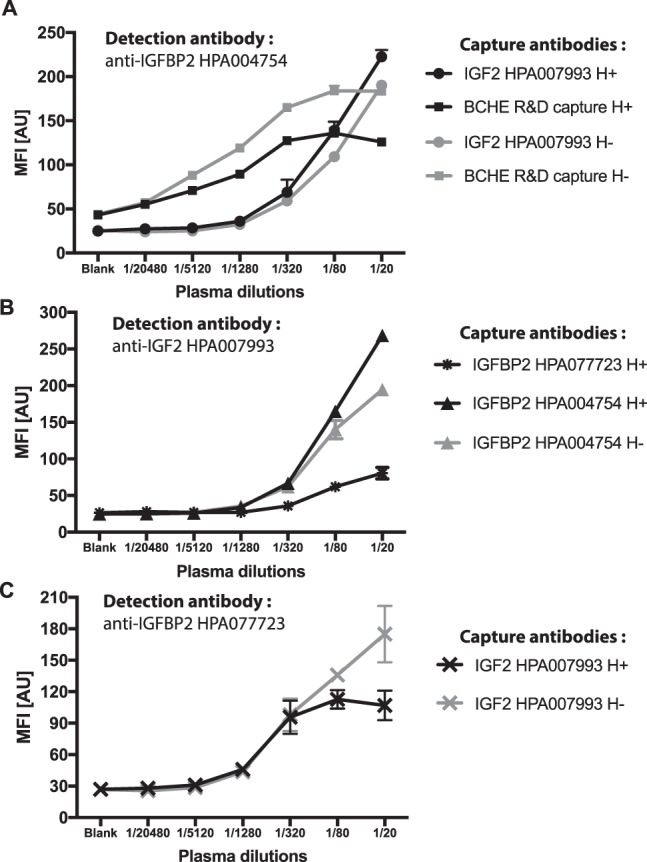
IGFBP2 protein interaction analysis by sandwich immunoassay. Dilution curves of plasma analyzed by sandwich assays using different combination of capture and detection antibodies. Dots represents mean value with standard error (SD) bars. In black, heat treated plasma (H+); in gray, untreated plasma (H−).

In order to provide further support for the possible interactions between the identified proteins, we conducted multiplexed sandwich assays. Here, recombinant IGFBP2, IFG1, IGF2 and BCHE were analyzed in a concentration dependent manner to annotate the assays’ functionality and target specificity (Table 3) and to confirm the selectivity of the matched antibody pairs in plasma. Then, we investigated if even pairs of antibodies with a different selectivity revealed plasma concentration dependent results. As shown in Fig. 4, we found pairs of antibodies with mixed specificity in the following capture-detection configurations: IGFBP2-IGF2, IGF2-IGFBP2 as well as BCHE-IGFBP2. For IGF1 and IGF2 antibody pairs, it was not possible to obtain a dilution curve with the respective recombinant proteins in solution, but they were functional in plasma (S-Fig. 9C,D,M,N,P). Also, IGF2-IGFBP2 and IGFBP2-IGF1 confirmed the presence of the previously known complex IGFBP2-IGF2 (Table 3, Fig. 4B,C). Antibody pairs for IGFBP2 and BCHE described a sample dilution depended trend with their corresponding intended recombinant proteins as well as in plasma (S-Fig. 9A,E,F,H,I). Since we did not observe cross-reactivity towards these two proteins with other antibodies in the assay (Table 3), the functional antibody pair BCHE-IGFBP2 supports the indications provided by IP, which pointed at a physical interaction between these two proteins in plasma (Fig. 4A). To further strengthen this observation, the use of an inverted assay configuration (IGFBP2-BCHE) and an assay including additional IGFBP2 antibodies, such as HPA077723 (Fig. 3B), would be preferred. Even though it was not possible to confirm a physical BCHE-IGF1 interaction, our data suggested that an interaction between BCHE and IGF1 or IGF2 could involve also IGFBP2 forming a larger complex built on IGFBP2-IGF2 (or IGF1)-BCHE (Fig. 4A). We acknowledge that not all antibodies allowed building mixed sandwich pairs with the chosen assay protocol, and above all, in the presence of protein complexes. HPA004754 and HPA077723 were raised against two different epitopes of IGFBP2, hence this could explain their different performance as either capture and detection antibody. HPA004754 was though functional as capture and detection antibody both using heat-treated and untreated plasma for the detection of IGFBP2, as well as in combination with anti-BCHE (S-Fig. 9B,G). HPA077723 was not functional with anti-BCHE either as a capture or detection antibody. This suggested that the binding of one antibody might hinder the binding other antibody to a complex of IGFBP2-BCHE. Further investigations could investigate if this incompatibility was due to the proximity of the two binding sites or other steric effects such as epitope accessibility of a captured complex.

**Table 3:** Antibody pairs tested in plasma and with recombinant proteins.

Samples	Detection Antibodies	Capture Antibodies				
		anti-IGFBP2	anti-IGF2	anti-BCHE	anti-IGF1	anti-DERA
Plasma	anti-IGFBP2	**+**	**+**	**+**	**−**	**−**
	anti-IGF2	**+**	**+**	**−**	**−**	**−**
	anti-BCHE	**−**	**−**	**+**	**−**	**−**
	anti-IGF1	**−**	**−**	**−**	**+**	**−**
	anti-DERA	**−**	**−**	**−**	**−**	**−**
Rec-IGFBP2	anti-IGFBP2	**+**	**−**	**−**	**−**	**−**
Rec-IGF2	anti-IGF2	**−**	**−**	**−**	**−**	**−**
Rec-BCHE	anti-BCHE	**−**	**−**	**+**	**−**	**−**
Rec-IGF1	anti-IGF1	**−**	**−**	**−**	**−**	**−**

Annotation (**+**): Trends from sample dilution assays were obtained with at least one combination of antibodies for the same protein either in heat-treated or untreated samples. Annotation (−): No sample concentration dependent data was obtained. (Details regarding each single pair are listed in Supplementary Excel Sheet “Reagents_Lot_Numbers”. Catalog numbers of the functional pairs are indicated in Fig. 4 and S-Fig. 9).

### OFF-target enrichment category

At last, we will discuss off-target enrichments. Here the abundance of the off-target over the intended analyte is most likely the main reason for failing to enrich the expected target in plasma. As the community is starting to acknowledge the fact that the performance of antibodies is indeed sample context and application dependent, certifying which other proteins or off-targets are bound may still allow generating novel hypotheses given that these are followed-up and thoroughly validated by appropriate targeted analysis.

One example for selective off-target binding in plasma is presented by the antibody HPA004920, raised against matrix metalloproteinase 1 (MMP1). We classified this antibody as OFF-target in untreated plasma because it enriched mannose-binding protein C (MBL2; z = 8.3; ƒ = 12%) as well as MMP3 (z = 6.8; ƒ = 6%) in the IP assays (Fig. 3A). As described above for CCL16 and SERPINA4, also here a 53% sequences similarity between the intended target (MMP1) and the off-target (MMP3) exists (S-Fig. 10B) and an interaction between these two proteins has been predicted (S-Fig. 10A). The other off-target MBL2 and MMP1 have only a 10% sequence similarity (S-Fig. 10C). MBL2 is though estimated to be present at 1.7 µg/ml in the blood circulation, hence it is almost 1000x more abundant than MMP1 ([c] = 1.1 ng/ml) and MMP3 ([c] = 0.5 ng/ml)^14^. MBL2 has also been described to reside in a complex with the MBL-associated serine protease (MASP)^31^, and its collagen-like domain may serve as a substrate for MMPs to nest in. Studies on MBL mutations suggest that MMPs may be involved in physiological regulation of MBL levels^32^. This could explain the presence of MBL2-MMP3 complexes in plasma.

## Conclusion

In summary, this study describes a resource that was built from proteins enriched from plasma and it was applied for the determination of antibody selectivity in plasma. The antibodies analyzed in this study include IgGs derived from different species and both polyclonal and monoclonal binders. We have conducted >400 IP assays in plasma and built a library of proteins with their frequencies of identification in plasma. Constructed on the systematic analysis of 157 antibodies, we described the occurrence of common proteins, denoted plasma background. This allowed us to determine the selective capture of endogenous proteins in plasma and their protein interactions via the z-scores. Some of these antibodies had been was used for exploratory plasma analysis on bead arrays and the development of immunoassays, where either heat-treated or untreated plasma served as samples.

Our approach, which we also compared with Western blot (Supplementary Section 2.12, S-Table 1), could serve as a valuable method to narrow down larger numbers of antibodies by determining which antibodies bind to their endogenous protein of interest. It can also provide information about interference from off-target binding events as well as possible proteins interactions. The intention behind using z-scores was to apply a measure for protein enrichment that would identify those proteins that were enriched above the large number of commonly detected background contaminants. The background was comprised of mostly abundant proteins that possibly bound to the antibody-coupled beads mainly due to their high concentration. Proteins often enriched in heat-treated plasma were C4A, TBC1D10C, MASP1 and PRRT3, while in untreated plasma EFEMP1, FBLN1, CAPZA2 and TTN were more frequently enriched (S-Fig. 8A,B). We also found that only a few proteins were commonly enriched when comparing more than 3 antibodies raised towards a common target.

This concept may though not yet fully elucidate (i) if the antibodies bind to proteins as full-length or fragments, (ii) if the antibodies will be functional in pairs in sandwich assays, (iii) how potential protein interactors and off-targets compete with on-target binding, and (iv) how contaminations from passenger antigens affect the assay’s selectivity and sensitivity. For antibodies annotated for ON-target and CO-target binding, further investigations are required to clarify the technical aspects mentioned above and to expand on the biological implication of protein interactions in plasma. While targeted fit-for-purpose experiments to further assess the protein complexes should include dose-response-curves using dilutions of plasma, these assays should preferentially be coupled to quantitative mass spectrometry analysis that rely on a set of peptides identified from the potential co-targets and/or contaminants. Nevertheless, IP-MS is also an informative approach for the identification of paired antibodies to study protein complexes in plasma by sandwich assays, where each antibody targets a different protein.

The presented study builds one foundation towards a more detailed assessment of the utility of antibodies for plasma proteomics assays, and may contribute to the development and application of more specific, robust and reliable immunoassays that can use mass spectrometry or other means of detection^1^.

## Methods

### Antibody coupling to magnetic beads

Covalent coupling of antibody to magnetic beads (MagPlex, Luminex Corp.) was performed as previously described^23^ using Sulfo-N-hydroxysulfosuccinimide and ethyl-carbodiimide (both Thermo). Each antibody (1.6 µg) was diluted in MES buffer with 500 000 beads and incubated for 2 h at room temperature, beads were subsequently washed and stored in blocking buffer at 4 °C.

### Immunocapture-mass spectrometry

Aliquots of 0.5 ml from each of the plasma pools (Seralab) were stored in cryogenic vials at −80 °C and thawed at 4 °C before use. Catalog and lot numbers are listed in S-Excel Table sheet “Experimental Batches”. Plasma (100 µl) was diluted 1:10 in assay buffer containing PVA (0.5% w/v), PVP (0.8% w/v), casein (0.1% w/v, all Sigma-Aldrich) and 10% of rabbit IgG (Bethyl Laboratories, Inc.). Diluted plasma and antibody-coupled beads were incubated overnight on a rotation shaker at 23 °C. Samples undergoing heat treatment were incubated for 30 min at 56 °C in water bath, before being combined with beads and incubated overnight. Using a magnetic bead handler (KingFisher™ Flex Magnetic Particle Processors, Thermo Scientific), beads were separated from the sample, washed with 0.03% Chaps in PBS and re-suspended in digestion buffer containing ammonium bicarbonate (50 mM) and sodium deoxycholate (0.25%). Proteins were reduced with DTT (1 mM) at 56 °C for 30 min, and alkylated by iodoacetamide (4 mM, all Sigma-Aldrich), at RT in the dark for 30 min. Alkylation was quenched adding 1 mM DTT. Proteins were digested using a mixture of Trypsin and LysC at 1:25 trypsin-to-protein ratio (Promega, USA) overnight at 37 °C. Enzyme inactivation and sodium deoxycholate precipitation was obtained adding 0.005% TFA. Peptides were then separated from beads, dried and re-suspended in solvent A containing 3% acetonitrile (ACN) and 0.1% formic acid (FA).

### LC-MS/MS

MS analysis was performed using a Q-Exactive HF (Thermo) operated in a data dependent mode, equipped with an Ultimate 3000 RSLC nanosystem, Dionex). Samples were injected into a C18 guard desalting column (Acclaim pepmap 100, 75 µm × 2 cm, nanoViper, P/N 164535, Thermo) and then into a 50 cm × 75 μm ID Easy spray analytical column packed with 2 μm C18 (EASY-Spray C18 P/N ES803, Thermo) for RPLC. Elution was performed in a linear gradient of Buffer B (90% ACN, 5% DMSO, 0.1% FA) from 3% to 43% in 50 min at 250 nL/min. Buffer A for the chromatography wa: 90% water, 5% ACN, 5% DMSO, 0.1% FA. Buffer B was increased stepwise to 45% in 5 min, then to 99% in 2 min, and then held for 10 min. Full MS scan (300–1600 m/z) proceeded at resolution of 60,000. Precursors were isolated with a width of 2 m/z and listed for exclusion for 60 s. The top five most abundant ions were selected for higher energy collision dissociation (HCD). Single and unassigned charge states were rejected from precursor selection. In MS/MS, a max ion injection time of 250 ms and AGC target of 1E5 were applied.

### Data processing

Shotgun MS data search was performed on MaxQuant (v1.5.3.30)^41^ using the integrated algorithm MaxLFQ. Spectra were searched against a human protein database from Uniprot (accessed on 03/17/2016, Canonical and Isoforms, 20,198 hits customized adding sequences of immunoglobulins chain C from rabbit, rat and mouse, LysC (PSEAE) and Trypsin (PIG). Settings allowed for two missing cleavages, methionine oxidation and N-term acetylation as variable modification and cysteine carbamidomethylation as fixed modification. In order to conduct a Label Free Quantification (LFQ) analysis with “delayed normalization”, we applied the “Fast LFQ” and “match between runs” functions of MaxQuant and set parameters to three minimum number of neighbors, and six average number of neighbors. Raw data produced to assess experimental conditions were analyzed using MaxQuant but excluding the function for LFQ.

The total data set was built on 465 unique data files including those from replicated injections. These replicated injections were performed for some IPs if the instrument did not perform optimally. When two technical replicates were available for the same IP, we excluded the replicate with the lower median LFQ intensity (data not shown). We included 414 IPs representing independent incubations of the 153 antibodies and control antibodies performed in independent incubations with EDTA plasma (biological replicates). The number of replicates per antibody is indicated S-Excel Tables.

No technical replicate (= replicated injections) were included for data analysis. Due to reasons related to costs, sample and reagent consumption, it was not possible to conduct the same number of replicates for all the antibodies and controls. A minimum number of two incubations were performed for each antibody. The different numbers of replicates reflect a limitation when validating those antibodies from which only low quantities are available. All raw files from the 414 unique IPs including replicate IPs for 153 antibodies plus negative controls and bare bead (S-Excel Tables, sheet “products and lot numbers”) were analyzed in a single MaxQuant session.

From a first data matrix of 1518 protein identifiers for 414 IPs, we excluded proteins from the analysis that we considered as contaminants: (i) proteins belonging to the list of contaminants in MaxQuant that do not belong to Homo sapiens, (ii) Ig gamma chain C region from Oryctolagus cuniculus (Rabbit) because we purposely add rabbit IgG to the assay buffer in order to reduce binding to rabbit IgG on the beads, as well as (iii) human Immunoglobulin variable chains. The final data matrix considered missing values as missing not at random (MNAR)^42^ but missing because of concentrations below the limit of detection (LOD). We therefore used min = 0 as minimum detected intensity (Single-value imputation approach). Consequently, LFQ = 0 were substituted to LFQ = 1 to allow for log10-transformation of the data. A data matrix with 1313 proteins related to 414 IPs were then used for further analysis.

### Data analysis

Using the processed data, we calculated the z-scores. First, we divided the data frame of 1313 proteins and 414 IPs into two parts containing 276 IPs with heat treated plasma and 138 IPs performed with untreated plasma. Using this data, we determined the frequency of identification (ƒ) for each protein by dividing the number of times a protein was detected (LFQ > 0) in the total number of IPs.

For each protein present in either of the two data sets, we then calculated an average LFQ intensity (µp) and determined the standard deviation of these LFQ intensities (sp), hence using all of the either 276 or 138 IPs. For each protein identified in the two data sets, we calculated the z-score according to the following formula:

$$zx\,in\,Ab1=(LFQintx-{\mu }p)/sp$$

where zx is the z-score for a protein x identified in each of the IPs performed for one of the 153 antibodies analyzed (Ab1), and LFQint the intensity of a detected protein. If biological replicates of IPs for specific antibodies were available, we merged the data into one by calculating the average of LFQint of these replicates and we only considered proteins identified in all replicates and disregarded all others.

This means that each protein will receive an antibody-related z-score. Proteins were considered enriched by an antibody when associated to a z-score ≥ 3. To visualize the enriched proteins for each antibody, z-scores and LFQint values were plotted as x and y axes and proteins found above the set threshold were annotated accordingly (Fig. 3 and S-Fig. 2B).

Data analysis and representation was performed with R^43^. Data analysis summary is represented in S-Fig. 11. Alignments were performed using the Clustal Omega program available at EMBL-EBI^44^. GO enrichment system was performed using the PANTHER Classification System (http://pantherdb.org/). Comparison of GO terms was conducted using ToppCluster (https://toppcluster.cchmc.org/), regarding Bonferroni corrected p-values < 0.01 as significant.

### Sandwich immunoassay

The capture antibodies towards IGFBP2, IGF1, IGF2, DERA, BCHE, and rabbit-Immunoglobulin G (rIgG) and mouse-IgG, as negative controls (S-Excel Table, sheet: “Reagents Lot numbers”), were covalently coupled to color-coded magnetic beads and analyzed in Luminex Platform as previously described^45^, using on-bead labeling for the detection antibodies^46^. The antibodies were tested in different combination of capture and detection pairs (S-Excel Table).

EDTA plasma was thawed on ice and centrifuged for 1 min at 2000 rpm and diluted from 1:20 following 4-fold dilutions in PVX casein (PVXC) buffer 10% rabbit IgG. The dilution series consisted of 6 points in duplicate and plasma was either heat-treated at 56 °C for 30 min or left on ice for 30 min. The plasma was subsequently incubated with the antibody-coupled beads overnight.

The recombinant proteins IGFBP2 and IGF1 were produced as full-length version in CHO cells^47^ and were a kind gift from Hanna Tegel and Johan Rockberg (AlbaNova University Center, KTH). IGF-II (R&D systems, catalog # 292-G2-050, lot DS2416011) and BCHE (DuoSet kit R&D systems, Catalog # DY6137-05, lot # 1387842) were commercially available. IGFBP2 and IGF2 were diluted in buffer from 500 ng/mL following 3-fold dilutions, IGF1 from 12000 pg/mL following 3-fold dilutions and BCHE 10000 pg/mL following 2-fold dilutions. The detection antibodies were applied at 1 µg/mL for HPA antibodies or 25 ng/mL anti-BCHE (R&D systems) for 90 min. Streptavidin-R-phycoerythrin (R-PE) conjugate (Life Technologies; SA10044) was used for the fluorescence read out in FlexMap3D (Luminex Corp.).

## Supplementary information

SUPPLEMENTARY INFORMATION

SUPPLEMENTARY EXCEL TABLES
