# Supplementary material for: Systematic assessment of antibody selectivity in plasma based on a resource of enrichment profiles
Source: Sci Rep. 2019 Jun 6;9:8324. doi: 10.1038/s41598-019-43552-5 (PMC6554399; doi:10.1038/s41598-019-43552-5)
Supplement: Supplementary file 1 — SUPPLEMENTARY INFORMATION [file 41598_2019_43552_MOESM1_ESM.docx]

**SUPPLEMENTARY INFORMATION**

**Systematic assessment of antibody selectivity in plasma based on a resource of enrichment profiles.**

Claudia Fredolini ^ǂ^, Sanna Byström ^ǂϷ^, Laura Sanchez-Rivera ^ǂϷ^, Marina Ioannou ^ǂ^, Davide Tamburro ^¥^, Fredrik Pontén ^#^, Rui M. Branca ^¥^, Peter Nilsson^ǂ^, Janne Lehtiö ^¥^ and Jochen M. Schwenk ^ǂ,*^

^ǂ^ Division of Affinity Proteomics, Science for Life Laboratory, Department of Protein Science, KTH - Royal Institute of Technology, 171 21 Solna, Sweden

^¥^Cancer Proteomics, Department of Oncology-Pathology, Science for Life Laboratory, Karolinska Institute, 171 21 Solna, Sweden

^#^ Department of Immunology, Genetics and Pathology, Science for Life Laboratory, Rudbeck Laboratory, Uppsala University, 751 85 Uppsala, Sweden.

^Ϸ^ These authors equally contributed to the work

*Corresponding author:

Jochen M Schwenk, Division of Affinity Proteomics, Science for Life Laboratory, Department of Protein Science, KTH - Royal Institute of Technology, Box 1031, 171 21 Solna, Sweden,

E: [jochen.schwenk@scilifelab.se](mailto:Jochen.schwenk@scilifelab.se); T: +46 (0)8 790 9869

Table of Contents

[1 MATERIALS 3](#_Toc4662199)

[1.1 Plasma samples 3](#_Toc4662200)

[1.2 Target selection 3](#_Toc4662201)

[2 SUPPLEMENTARY TEXT AND FIGURES 4](#_Toc4662202)

[2.1 Antibody and antigen coverage 4](#_Toc4662203)

[S-Fig 1A: Distribution of number of antibodies targeting each protein. 4](#_Toc4662204)

[S-Fig 1B: Location of identified peptides. 4](#_Toc4662205)

[2.2 Optimization and pilot study 5](#_Toc4662206)

[S-Fig 2A: Testing bead and plasma concentrations. 5](#_Toc4662207)

[S-Fig 2B: IP-MS enrichment profiles of pilot study 7](#_Toc4662208)

[2.3 Sample, heat and batch effects 8](#_Toc4662209)

[S-Fig 3A: Heat map of IP-MS data 8](#_Toc4662210)

[S-Fig 3B: PCA plot of IP-MS data 8](#_Toc4662211)

[2.4 Comparison of estimated plasma concentration, frequency, LFQ 9](#_Toc4662212)

[S-Fig 4A and 4B: Relationship between plasma concentration and frequency. 9](#_Toc4662213)

[S-Fig 4C, 4D and 4E: Relationship between plasma concentration, LFQ and z-scores. 9](#_Toc4662214)

[2.5 Assessment of proteins linked to sample heat-treatment 10](#_Toc4662215)

[S-Fig 5A. Comparison of LFQ protein intensities. 10](#_Toc4662216)

[S-Fig 5B: LFQ intensities for FGA, FGB, FGG in heat and un-heated samples 10](#_Toc4662217)

[S-Fig 5C: Z-scores for FGA, FGB, FGG in heat and un-heated samples 10](#_Toc4662218)

[2.6 Investigation of passenger proteins 12](#_Toc4662219)

[S-Fig 6: Detection of passenger proteins on beads. 12](#_Toc4662220)

[2.7 Interaction and homology analysis of co-enriched proteins. 13](#_Toc4662221)

[S-Fig 7A: Interaction networks of CCL16 and CCL18. 13](#_Toc4662222)

[S-Fig 7B: Sequence homology search for CCL16-CCL18. 13](#_Toc4662223)

[S-Fig 7C: Interaction networks of SERPINA4 and SERPINA6. 14](#_Toc4662224)

[S-Fig 7D: Sequence homology search for SERPINA4-SERPINA6 14](#_Toc4662225)

[2.8 Assessing the distribution of enriched proteins 15](#_Toc4662226)

[S-Fig 8A: Enrichment distribution. 15](#_Toc4662227)

[S-Fig 8B-C: Proteins detected in multiple IPs. 16](#_Toc4662228)

[S-Fig 8D: Defining LFQ cut-off level for consistent identifications. 17](#_Toc4662229)

[S-Fig 8E-H: Concordance of IGFBP2 enrichment profiles. 18](#_Toc4662230)

[2.9 Sandwich immunoassays 19](#_Toc4662231)

[S-Fig 9A-Q: Combinations of antibodies were tested in plasma. 19](#_Toc4662232)

[2.10 Interaction and homology analysis of enriched off-target proteins. 20](#_Toc4662233)

[S-Fig 10A: Interaction network of MMP1 and MMP3. 20](#_Toc4662234)

[S-Fig 10B: Sequence homology search for MMP1-MMP3. 20](#_Toc4662235)

[S-Fig 10C: Sequence homology search for MMP3-MBL2. 21](#_Toc4662236)

[2.11 Clarification of data analysis strategy 22](#_Toc4662237)

[S-Fig 11: Data analysis workflow. 22](#_Toc4662238)

[2.12 Antibodies evaluation by protein arrays, WB and IP. 23](#_Toc4662239)

[S-Table 1: Comparison of validation rate on WB and IP-MS. 24](#_Toc4662240)

[3 CONTENT OF SUPPLEMENTARY EXCEL TABLES 25](#_Toc4662241)

[4 SUPPLEMENTARY REFERENCES 26](#_Toc4662242)

# MATERIALS

## Plasma samples

Aliquots of 0.5 ml from each of the plasma pools were stored in cryogenic vials at -80ºC and thawed at 4ºC before use. Catalog and lot numbers are listed in **S-Excel Table sheet “Experimental Batches”**.

## Target selection

Information about target proteins their functions and involvement in diseases were collected through literature searches, Gene Ontology (GO, <http://www.geneontology.org/>), Human Protein Atlas (HPA, <http://www.proteinatlas.org/>), and Early Detection Research Network (EDRN, <https://edrn.nci.nih.gov/)>. Plasma protein abundances were obtained from the updated Plasma PeptideAtlas of 2017^1^ ([www.peptideatlas.org)](http://www.peptideatlas.org)). Proteins were classified using the following terms: (i) Cellular or Extracellular when the proteins appeared in one or more of the terms extracellular region (GO:0005576); extracellular space (GO:0005615); extracellular exosome (GO:0070062); proteinaceous extracellular matrix (GO:0005578); (see columns “GO CC (Cellular Compartment) Complete” and “Summary of GO CC” in the **S-Excel sheet** “Protein Annotation GO”). The list of antibodies and information was reported in **S-Excel Tables**. The study included 157 antibodies: There were 15 monoclonal antibodies (N=10 from R&D Systems; N=1 from HyTest Ltd.; N=3 from Atlas Antibodies and N=1 from SigmaAldrich) and 142 polyclonal antibodies from the Human Protein Atlas ^2^. In addition, normal rabbit IgG (Bethyl Laboratories), mouse IgG and rat IgG (both Santa Cruz Biotechnology) were included as references for IgG. Catalog and lot numbers are listed in **S-Excel Tables**. For antibodies obtained by the Human Protein Atlas, antibody IDs and lot numbers are identical. Four antibodies out of 157 were used only in the optimization of the protocol (see **S-Excel Table, sheet “antibodies_experim_annotation”**). Therefore only 153 antibodies were included for the z-scores calculation described below.

# SUPPLEMENTARY TEXT AND FIGURES

## Antibody and antigen coverage

#### S-Fig 1A: Distribution of number of antibodies targeting each protein.

The majority of proteins targeted in this study (80%, N=95) were investigated using one antibody. Twenty-five proteins (20%) were targeted by ≥ 2 antibodies.


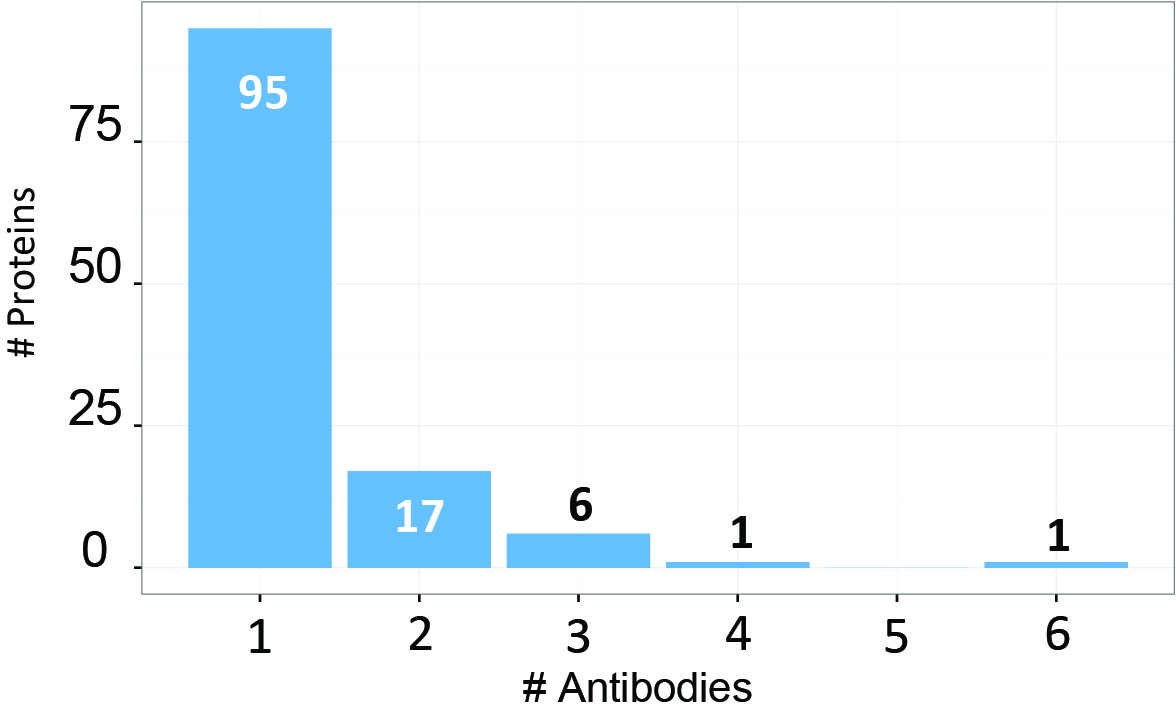


#### S-Fig 1B: Location of identified peptides.

Distribution of ON-target and CO-target antibodies based on the peptides identified for the intended target proteins are shown. Peptides representing parts of the used PrEST antigen sequence (in PrEST), peptides not covering the PrEST antigen (outside PrEST) or peptides identified inside as well as outside of the PrEST antigen sequence (both).

**
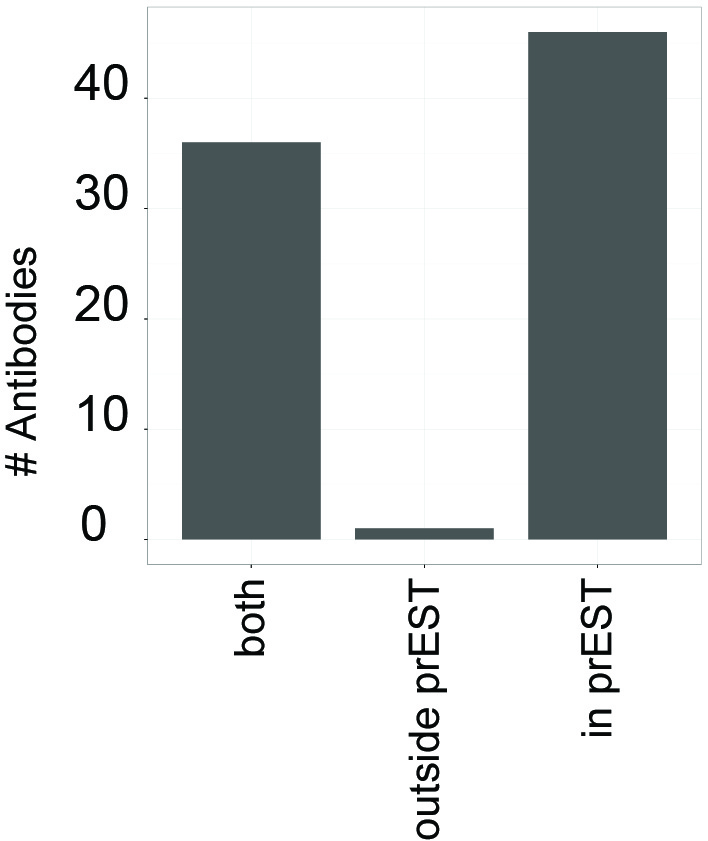
**

## Optimization and pilot study

Factors such as target protein concentration and properties of ionization of the peptides of interest would ideally require optimized analysis for each antibody/target. Nevertheless, we began with developing a procedure applicable to a broad range of antibodies and target proteins. In order to set up optimal experimental conditions, we evaluated technical aspects such as volume of neat plasma and amount of antibody. As expected, increasing the volume of plasma enhanced detection of low peptides belonging to low abundant proteins. For example interleukin 1 alpha (IL1A), with concentrations ~3 pg/mL in healthy human plasma ^3^, was detectable when 1 mL of plasma and 3.2 μg of antibody were applied (**S-Fig 2**). We established our protocol to enable the detection of proteins in a range of concentrations from µg/mL (C2) ^4^ to high pg/mL (KLK3) ^5^ ^6-9^.

#### S-Fig 2A: Testing bead and plasma concentrations.

Antibodies targeting known plasma proteins of different abundance (C2, CCL16, CST3, IL1A, KLK3, LCN2) were used to establish optimal experimental conditions for volume of plasma and number of beads coupled to antibody. In the axis legends, ‘p’ refers to the volume of plasma in µL; and ‘b’ to number of coupled beads (e.g. 500b = 500,000 beads = 1.6 µg of antibody).

**
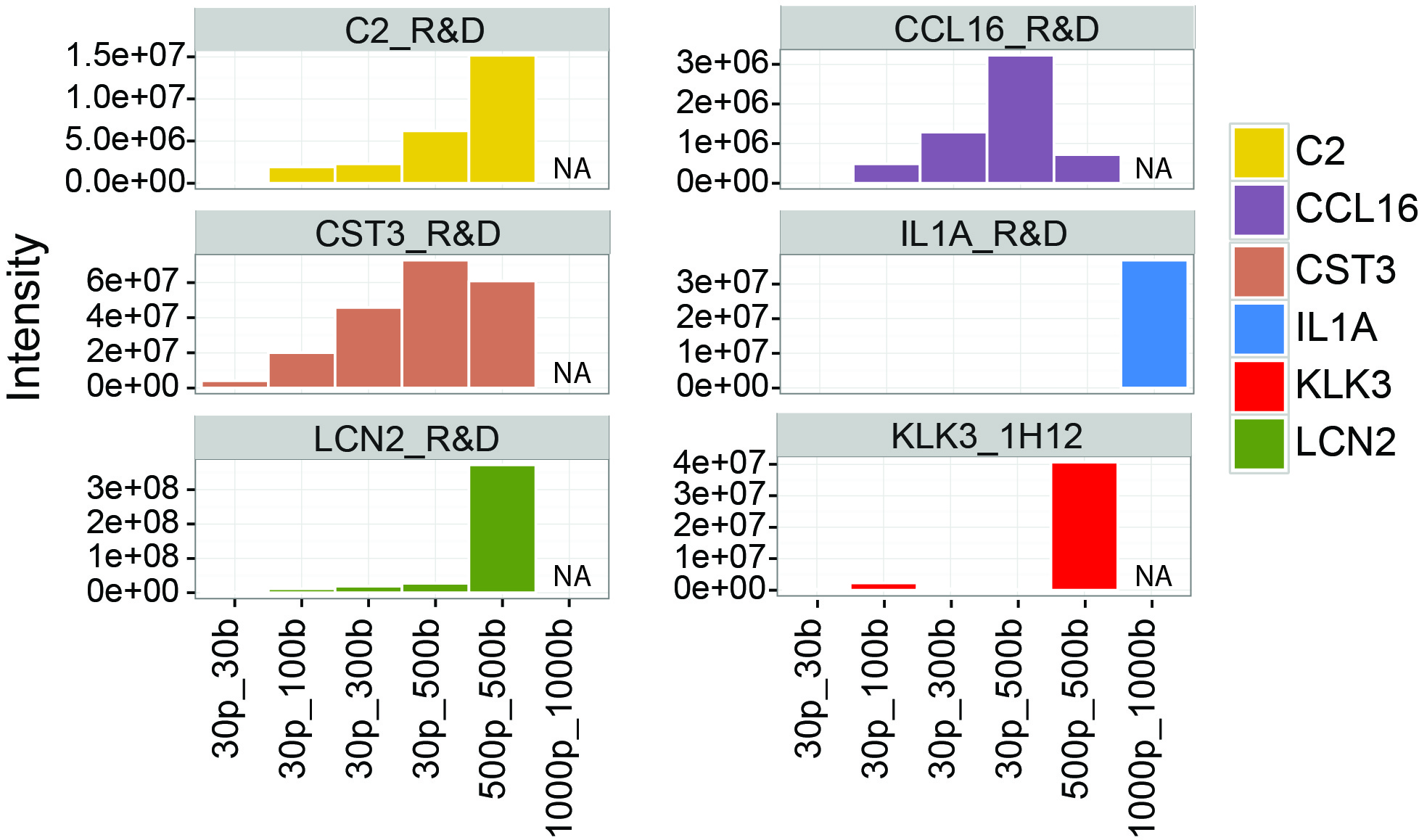
**

After the optimizations, we conducted a pilot study in which we compared the experimental conditions to reach an analytical sensitivity to detect plasma proteins and to assess the potential to discriminate between specifically captured or frequently observed proteins (denoted plasma background). A set of antibodies obtained from commercially available ELISA kits for plasma protein analysis were used to cover a plasma concentration range between μg/ml (CRP) to low pg/ml (IL1A). We tested combinations of different amounts of plasma (30-1000 µl) as well as increasing amounts of beads (30,000 - 1,000,000) to assess the detectability of the targets shown in S-Fig 1. We concluded that 100 µl of plasma and 500,000 beads were suitable for larger scaled investigations, enabling the detection within μg/ml and high pg/mL (e.g. KLK3 was detected in a pool of plasma obtained by mixing samples from healthy females and males, see S2B Figure and Text). For the detection of pg/ml plasma proteins, such as IL1A, the IP may require even larger amounts of sample and beads, which was found less suitable for large scale efforts. The LFQ intensities from the IPs were used to calculate z-scores and compare peptides from replicated assays. In the pilot, all the antibody profiles barely reached an enrichment threshold (z-score ≥ 3) when using only this limited set of assays (21) in the library for data analysis. Utilizing a larger library of plasma IP data, as introduced and described below, improved the number of expected target proteins with z-scores ≥ 3 (S-Fig 2).

#### S-Fig 2B: IP-MS enrichment profiles of pilot study

Data derived from IP assays with antibodies targeting the plasma proteins of C2, CCL16, CRP, CST3, NGAL and KLK3 (PSA) was analyzed using the pilot library limited to only these 21 IPs (left) as well as the library of more than 400 IP (right). In this experiment, 100 µl of plasma and 500,000 beads were applied.

## Sample, heat and batch effects

We observed that independent IPs performed in the same batch clustered together (**S-Fig 3A**). Parameters varying between independent batches IPs include different lots of reference plasma, trypsin, and analytical columns. Long-term drift in instrumental response, sample handling and sample heat-treatment may also add additional variability. We found that the main difference between the different assays was due to heat treatment of the samples. Indeed, despite experimental batches, assays using either heat treated or untreated plasma clustered together (**S-Fig 3** **A-B**). For this reason, we decide to analyze IPs with heat treated and not heat-treated plasma separately, in order to compare the enrichment profiles from the IPs with similar background.

#### **S-Fig 3A: Heat map of IP-MS data**

Two-ways hierarchical clustering analysis of the intensity values for the 1314 proteins identified (horizontal axes) in 414 immunoprecipitations (vertical axes). Clustering distance: "euclidean”; Clustering method: Ward. Cluster on theleft ofthe heat map represent (1) different experimental batches (dates are indicated accordingly to S-Excel Table Sheet “Experimental_Batches”and (2) sample heat treatment. On the right , color scale black to red is referred to the log10 (LFQ) intensity values.


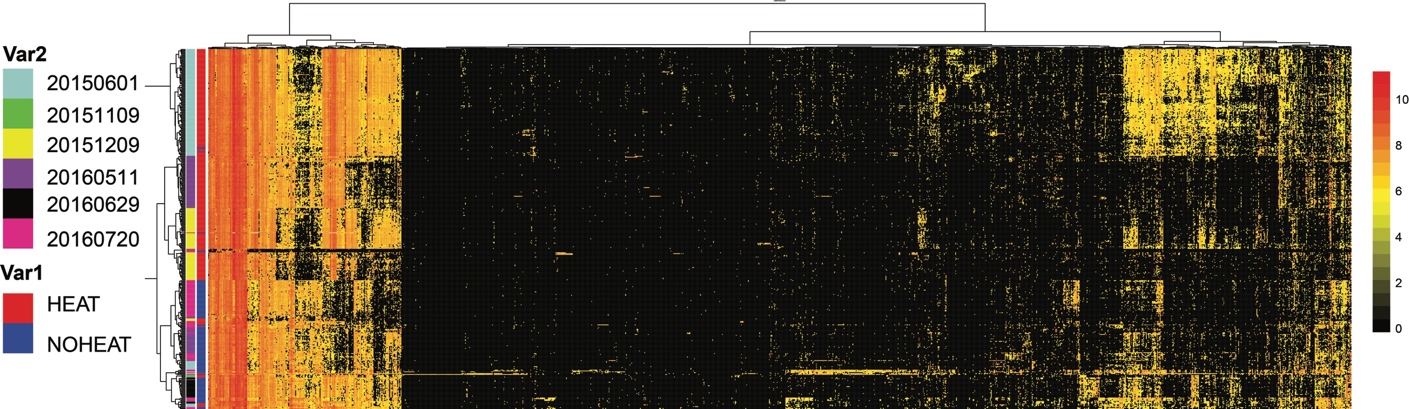


#### S-Fig 3B: PCA plot of IP-MS data

Representation of principal component analysis, dots colours highlight separated experimental batches.


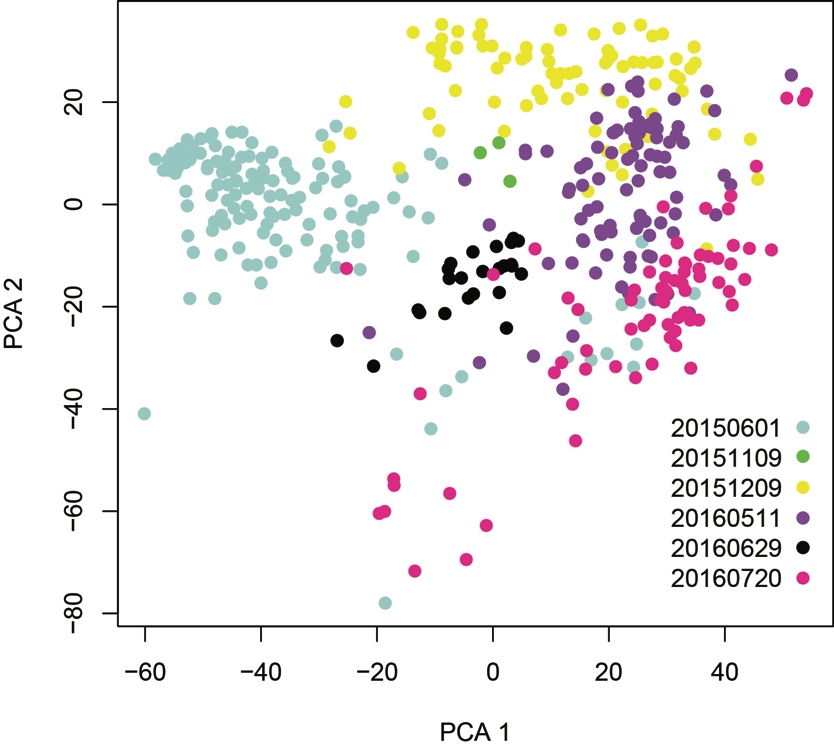


## Comparison of estimated plasma concentration, frequency, LFQ

We investigated the relation between the frequency of occurrence of 1313 proteins in untreated plasma (**S-Fig 4A**) and heat-treated plasma (**S-Fig 4B**) with the estimated plasma concentration from PeptideAtlas. There was a significant association (p-value < 2.2e-16) for both sample types while 293/1313 = 22% of the proteins (blue dots) were not found with a concentration estimation in PeptideAtlas. Similarly we investigated the relation between estimated concentration, LFQ value of intensity and z-scores (**S-Fig 4C-E**).

#### S-Fig 4A and 4B: Relationship between plasma concentration and frequency.

The estimated plasma concentrations are shown as [ng/ml] and frequency [%] for (**A**) untreated plasma and (**B**) heat treated plasma.


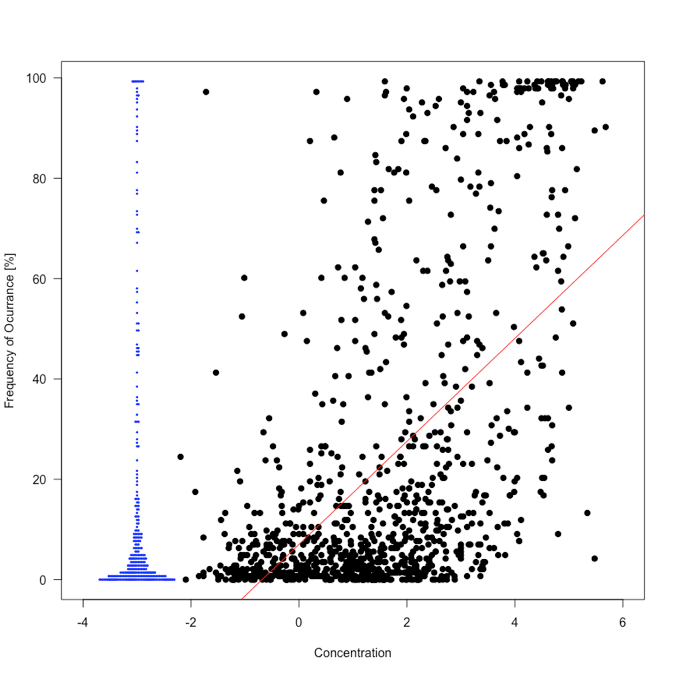

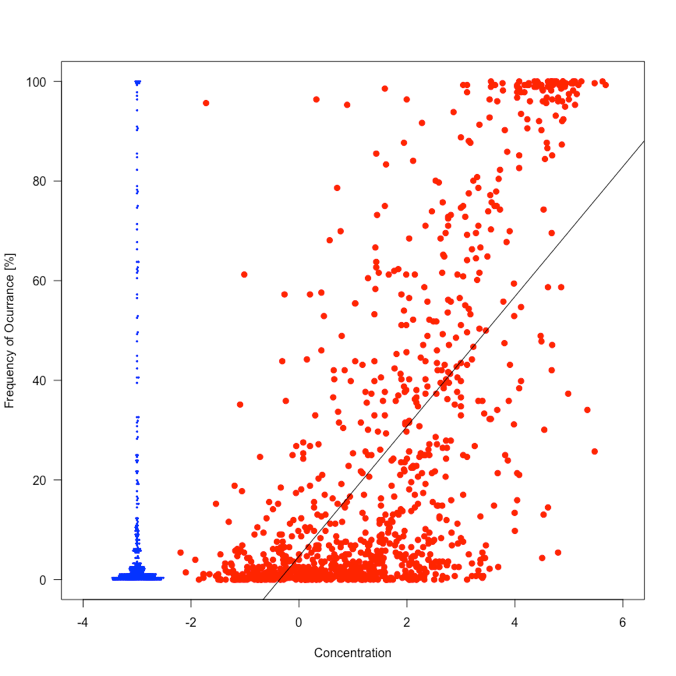


#### S-Fig 4C, 4D and 4E: Relationship between plasma concentration, LFQ and z-scores.

## Assessment of proteins linked to sample heat-treatment

IP-MS assays were performed in heat treated and in not heat-treated plasma. Fibrinogen alpha, beta and gamma chain (FGA, FGB, FGG) were strongly effected by heat-treatment.

#### S-Fig 5A. Comparison of LFQ protein intensities.

Comparison of LFQ intensities for protein identified in heat treated and hot heat treated samples. Circled in red high abundant plasma proteins which abundance in the background increases when plasma is heat treated.


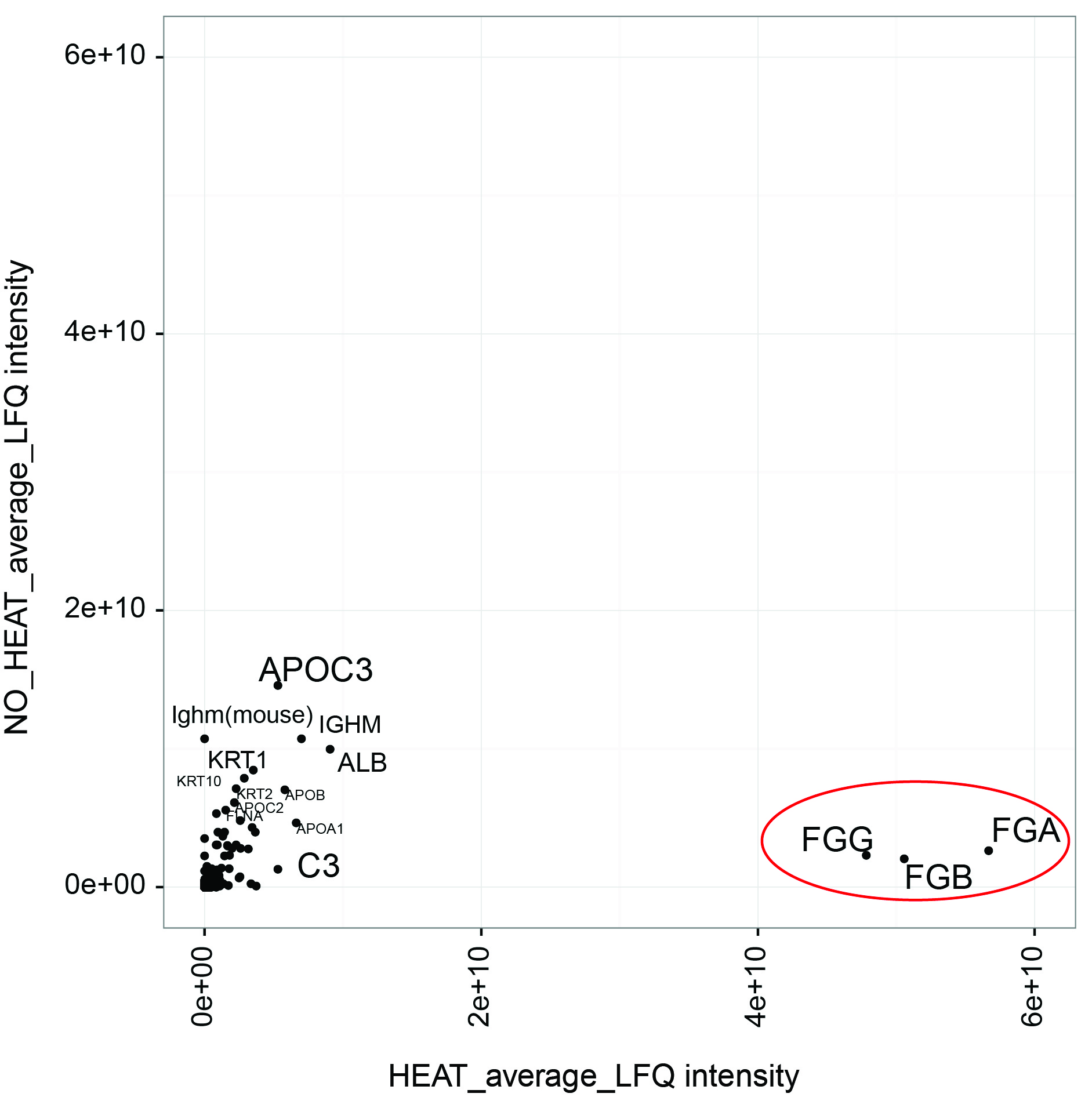


#### S-Fig 5B: LFQ intensities for FGA, FGB, FGG in heat and un-heated samples

#### S-Fig 5C: Z-scores for FGA, FGB, FGG in heat and un-heated samples

## Investigation of passenger proteins

We determined possible presence of antigens in the antibody solution. Antigens that were used during the affinity purification may have been co-eluted. Each antigen, denoted PrEST, consists of a tag (His_6_ and albumin binding protein ABP) and the region selected target protein ^10^. To detect the presence of the antigens, we chose an antibody specific to tag region (1:60,000; ^11^) and incubated antibody-coupled beads accordingly. Detection using an anti-IgY antibody carrying R-PE (1 µg/ml) we determined the cut-off as 10x SD + mean MFI obtained from beads carrying normal rabbit IgG, resulting in MFI = 135 AU. Out of 3,424 beads carrying 2,452 unique antibodies (**S-Fig 5**), 47 had been part of this IP-MS study. Applying the cut-off, 11% (390/3424) of the beads carrying 3% (77/2,452) of the antibodies indicated the presence of the antigen. Among these 77 binders , 11 were studied here (7%) , with only one binder from the ON-target category, nine binders from CO-target and also one OFF-target binder.

#### S-Fig 6: Detection of passenger proteins on beads.

Ranked distribution of the MFI levels obtained from the detection of possible passenger antigens on antibody coupled beads.


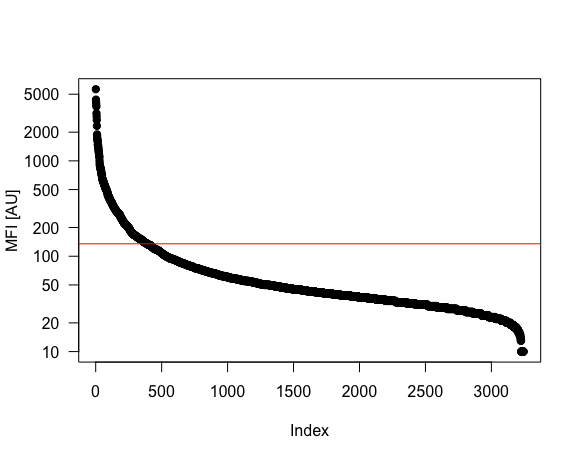


## Interaction and homology analysis of co-enriched proteins.

We observed co-enrichment of proteins for antibodies targeting CCL16 and SERPINA4. In the following, we describe the relationship and interactions of these with the observed off-targets by using String database V10.5 (20 interactors, 1st shell only) and sequence homology searches using CLUSTALO alignment.

#### S-Fig 7A: Interaction networks of CCL16 and CCL18.


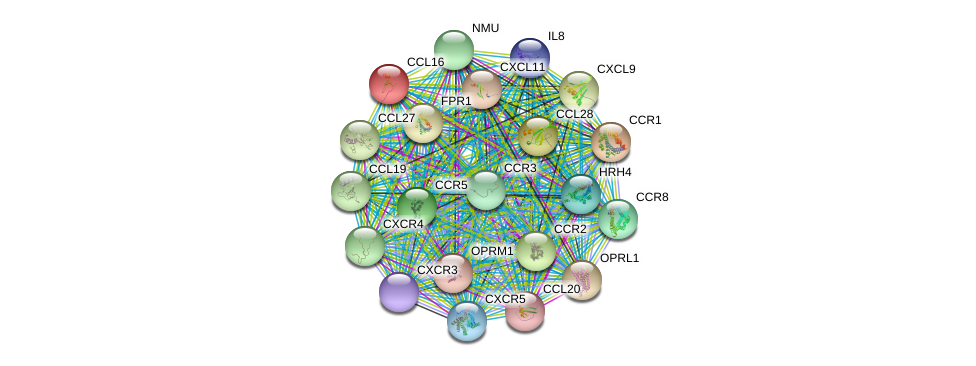

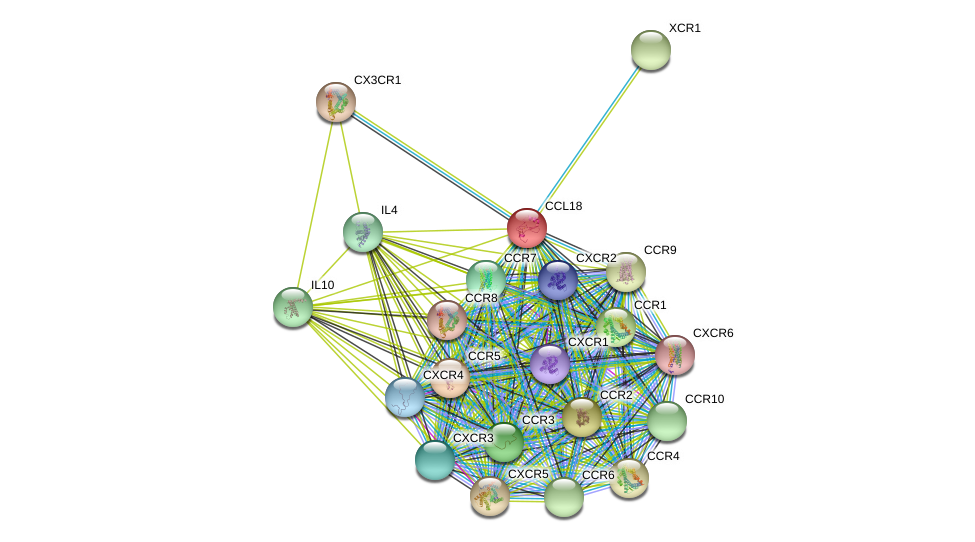


#### S-Fig 7B: Sequence homology search for CCL16-CCL18.

Alignment revealed a homology of 26.6%. The sequence used as antigen for the generation of the antibody is highlighted in yellow.

#### S-Fig 7C: Interaction networks of SERPINA4 and SERPINA6.


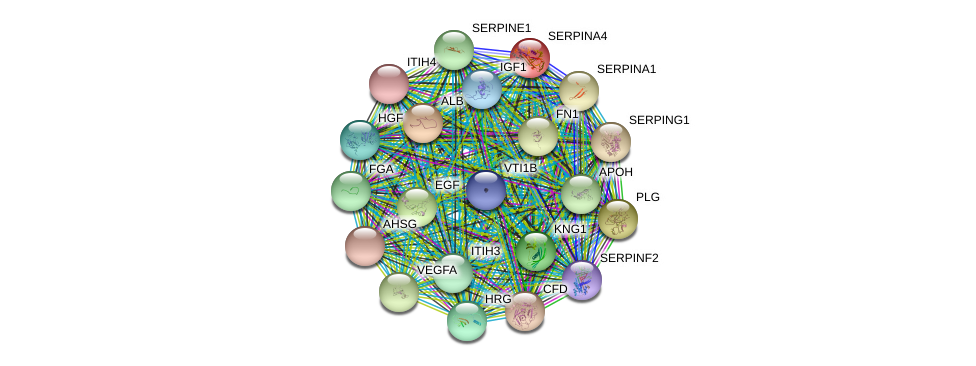

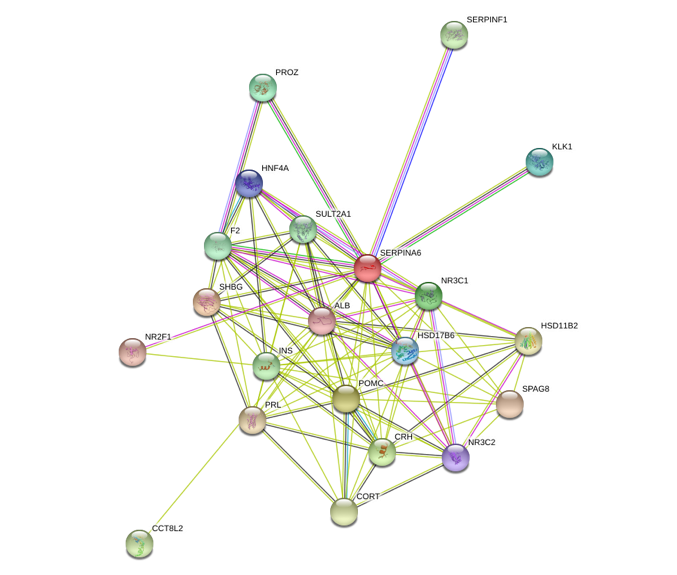


#### S-Fig 7D: Sequence homology search for SERPINA4-SERPINA6

Alignment revealed a homology of 40.0%. The sequence used as antigen for the generation of the antibody is highlighted in yellow.

## **Assessing the distribution of enriched proteins**

#### **S-Fig 8A: Enrichment distribution.**

Bar plot representation of the number of proteins to which a z-score ≥ 3 was assigned. Values of z-scores were rounded.


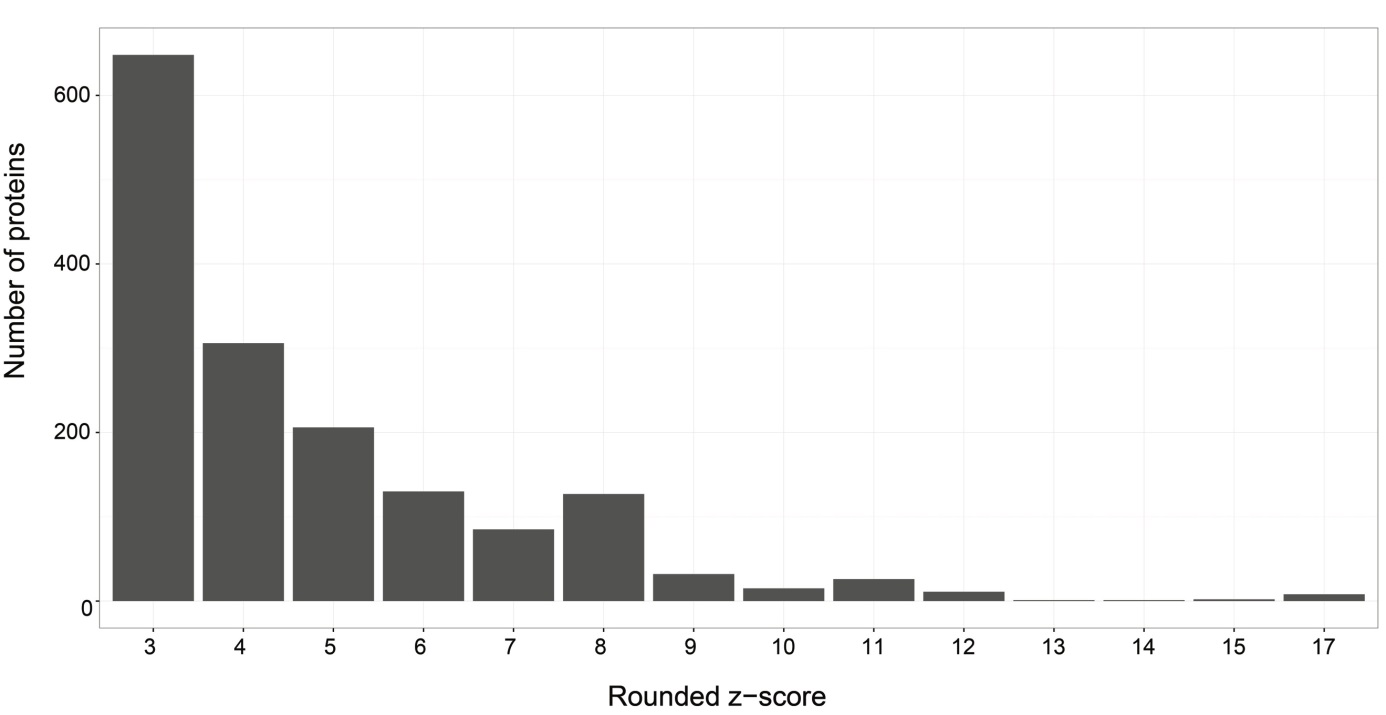


#### S-Fig 8B-C: Proteins detected in multiple IPs.

Bar plot represents those proteins enriched as CO-Target, OFF-Target and negative control by at least two antibodies in either heat-treated (**B**) or untreated (**C**) plasma.


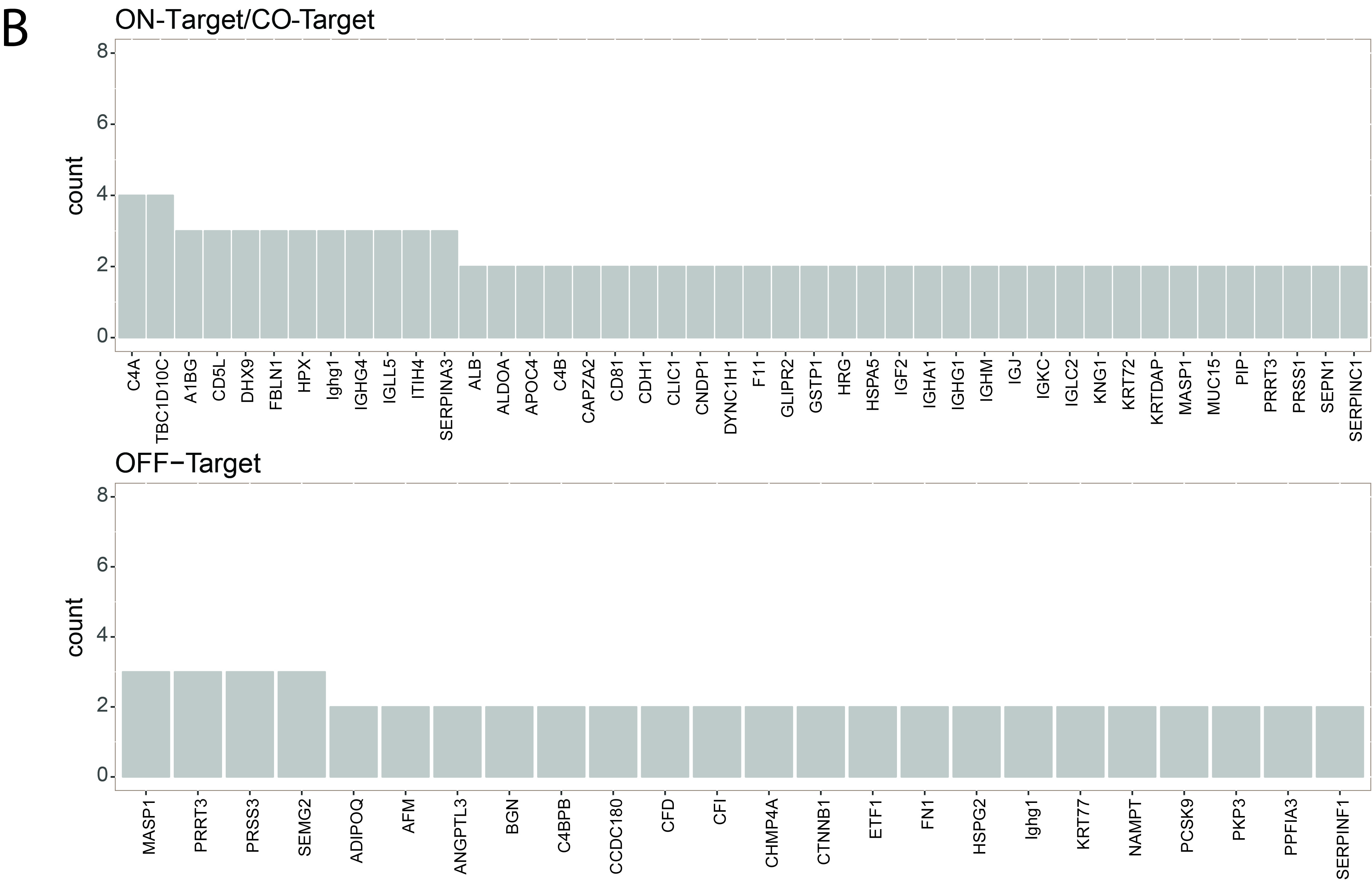


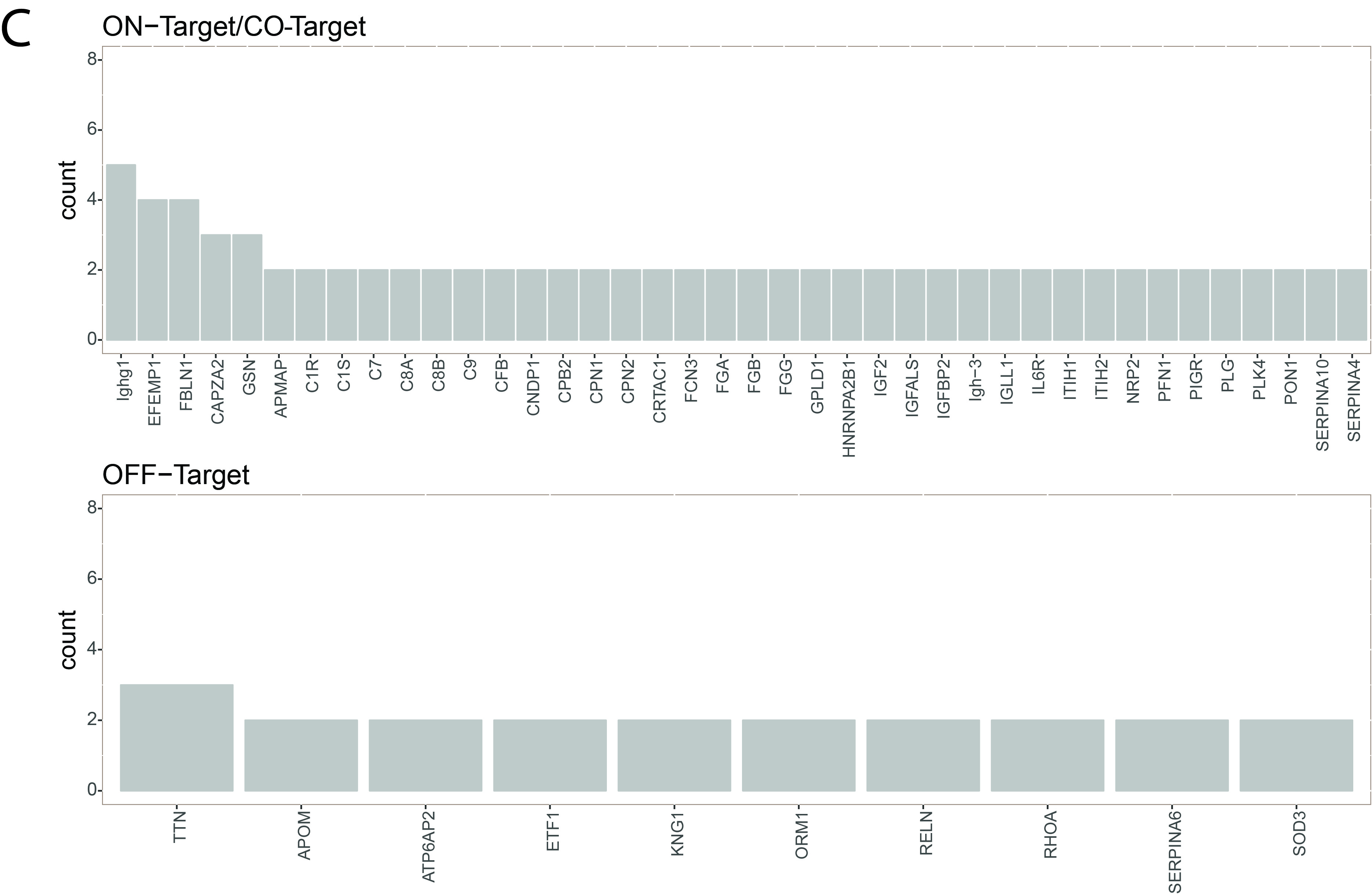


#### S-Fig 8D: Defining LFQ cut-off level for consistent identifications.

Defining a LFQ cut-off base on the Distribution of LFQ intensities associated to proteins identified in a single; duplicate and in triplicate experiments.


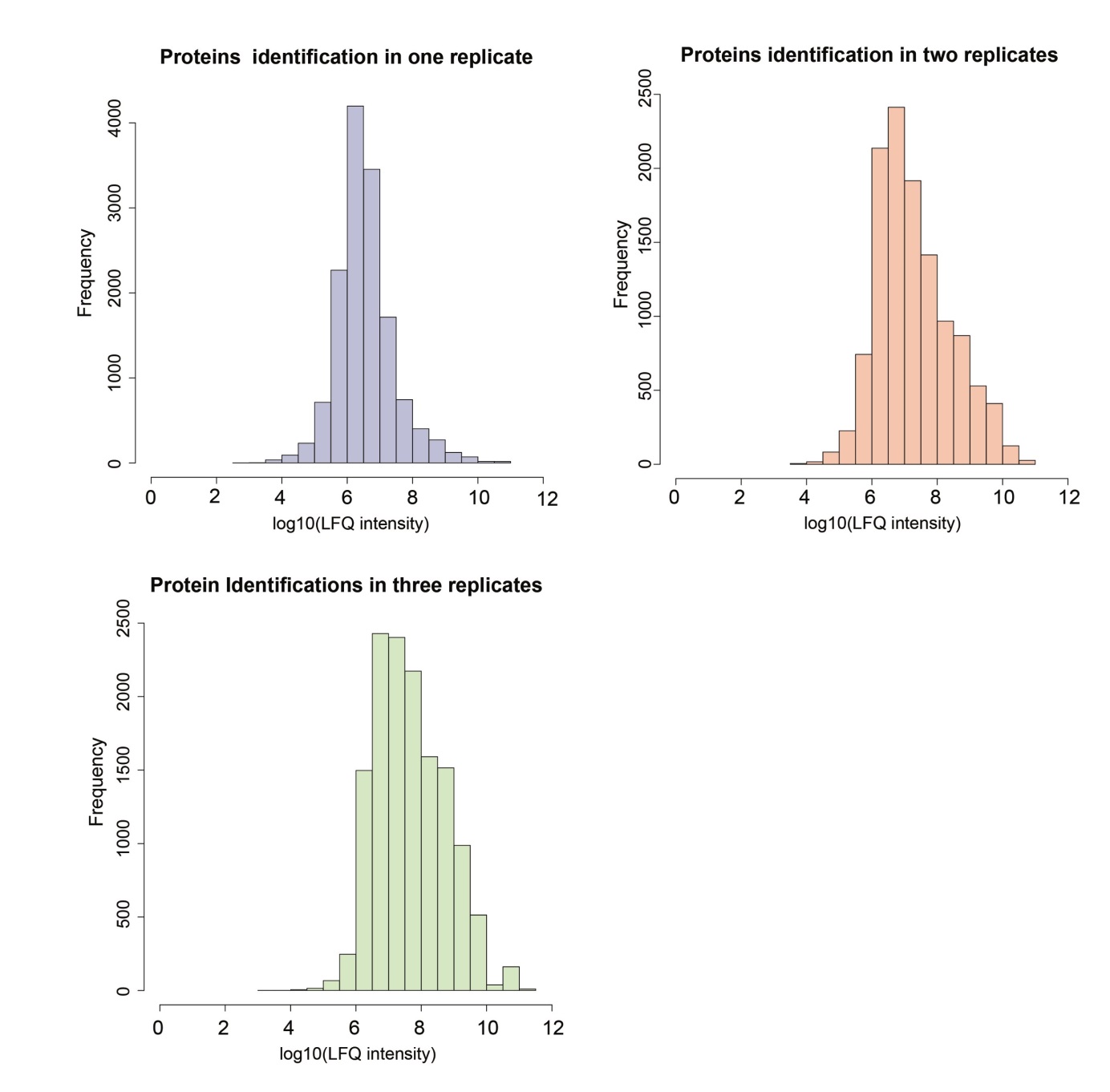


#### S-Fig 8E-H: Concordance of IGFBP2 enrichment profiles.

The scatterplots indicate the concordance of identified proteins between replicated assays performed with four different anti-IGFBP2 antibodies. Intra-batch replicates were compared for HPA004754 using heat-treated (F) and untreated plasma (G), respectively. Intra-batch replicates with HPA077723 were performed using untreated plasma (H). For HPA45140 (I), inter-batch assay replicates were performed on two separate occasions. The dashed red lines indicate a LFQ intensity of 10^7^.

**
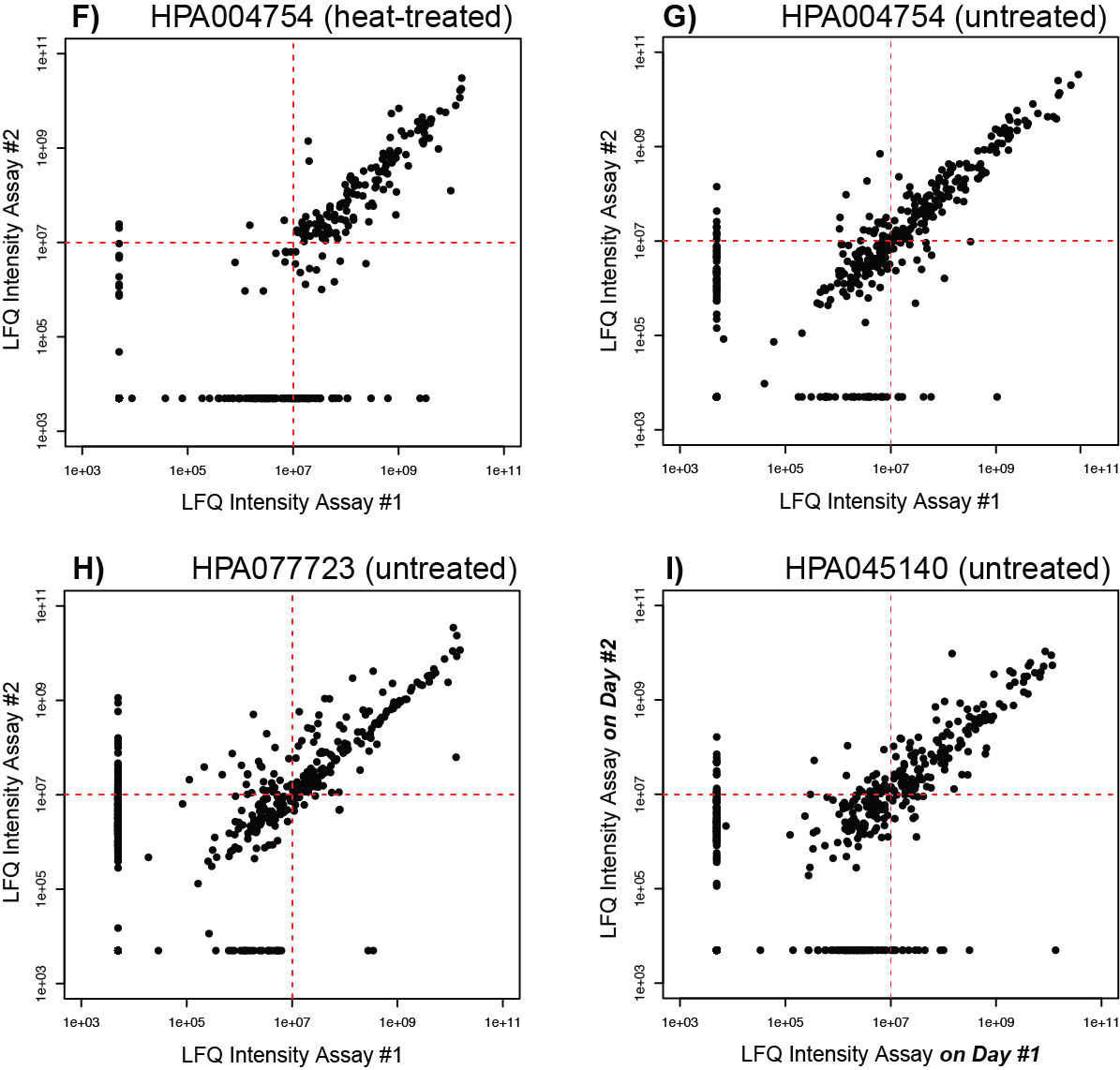
**

## Sandwich immunoassays

#### S-Fig 9A-Q: Combinations of antibodies were tested in plasma.

The MFI values of the corresponding pair of antibodies indicated for the EC50 plasma concentration. Headers of the plots indicate the detection antibody and on the x axis is the capture antibody. The data calculated is based on the mean values (±SD) of duplicate dilution curves. Dark grey (H+, heated plasma), Light grey (H-, untreated plasma).

**
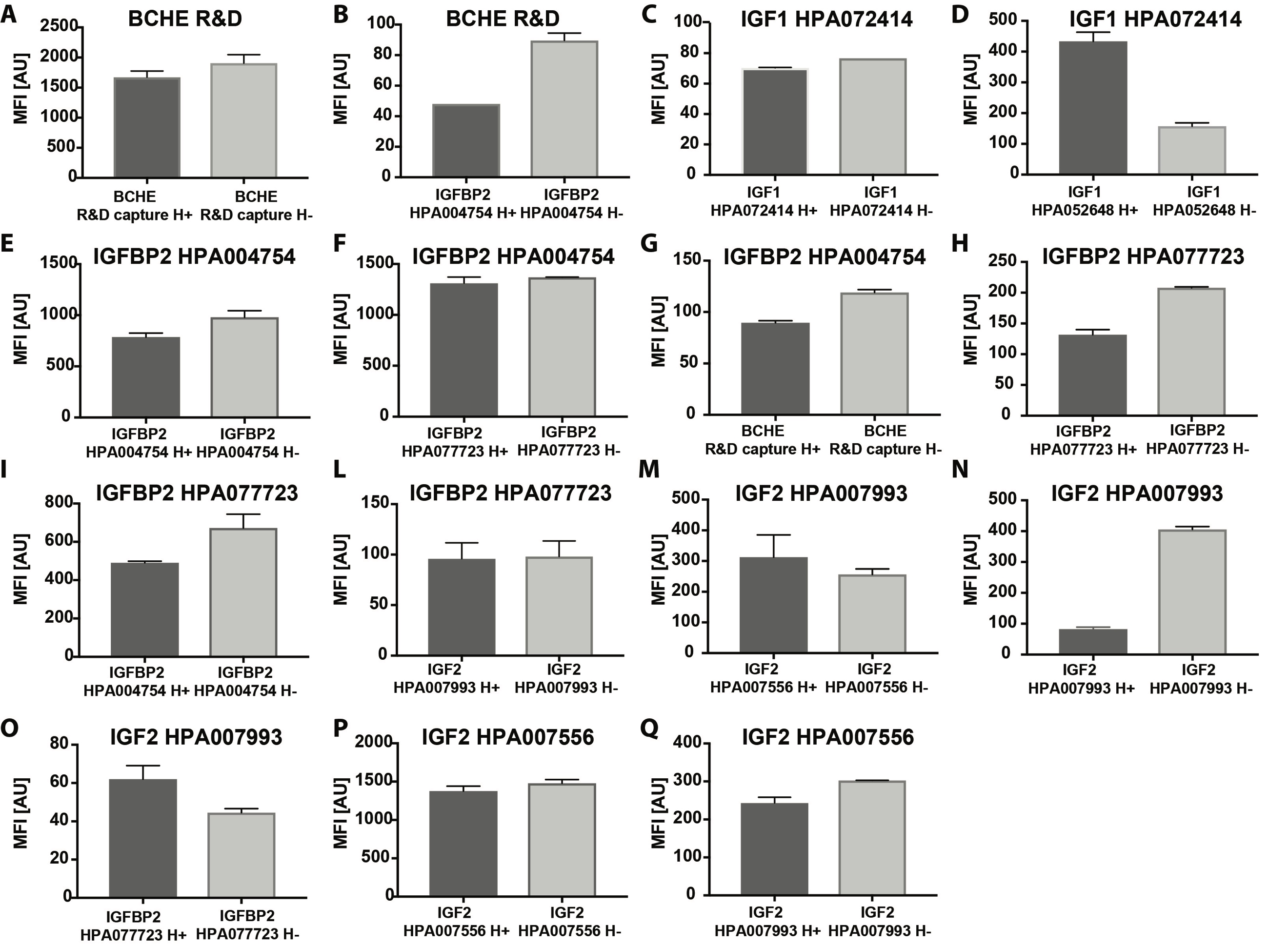
**

## Interaction and homology analysis of enriched off-target proteins.

We observed off-target enrichment of MMP3 when using an anti-MMP1 antibody. In the following, we describe the relationship and interactions of these with the observed off-targets by using String database V10.5 (20 interactors, 1st shell only) and sequence homology searches using CLUSTALO alignment.

#### S-Fig 10A: Interaction network of MMP1 and MMP3.


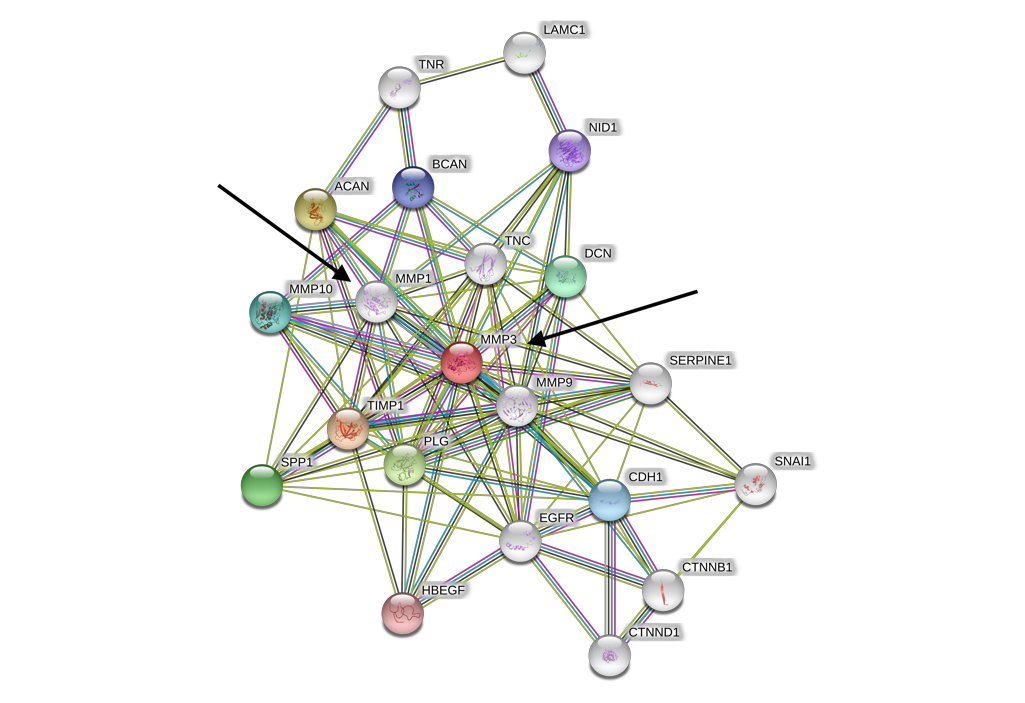


#### S-Fig 10B: Sequence homology search for MMP1-MMP3.

Alignment revealed a homology of 52.8 %. The sequence used as antigen for the generation of the antibody is highlighted in yellow.

#### S-Fig 10C: Sequence homology search for MMP3-MBL2.

Alignment revealed a homology of 9.981 %.

CLUSTAL O(1.2.4) multiple sequence alignment

SP|P08254|MMP3_HUMAN ---MKSLPILLLLCVAVCSAYPLDGAARGEDTSMNLVQKYLENYYDLKKDVKQFVRRKDS 57

SP|P11226|MBL2_HUMAN MSLFPSLPLLLLSMVAAS--YS-------------------------------------- 20

: ***:*** **.. *

SP|P08254|MMP3_HUMAN GPVVKKIREMQKFLGLEVTGKLDSDTLEVMRKPRCGVPDVGHFRTFPGIPKWRKTHLTYR 117

SP|P11226|MBL2_HUMAN ------------------------------------------------------------

SP|P08254|MMP3_HUMAN IVNYTPDLPKDAVDSAVEKALKVWEEVTPLTFSRLYEGEADIMISFAVREHGDFYPFDGP 177

SP|P11226|MBL2_HUMAN -----ETVTC-------E---------------------------------------DAQ 29

: * *.

SP|P08254|MMP3_HUMAN GNVLAHAYAPGPGINGDAHFDDDEQWTKDTTGTNLFLVAAHEIGHSLGLFHSANTEALMY 237

SP|P11226|MBL2_HUMAN KTCPAVIACSSPGINGFPGKDGRDGT-K-------------------------------- 56

. * . .***** *. : *

SP|P08254|MMP3_HUMAN PLYHSLTDLTRFRLSQDDINGIQSLYGPPPDSP--ETPLVPTEPV----PPEPGTPANCD 291

SP|P11226|MBL2_HUMAN ------------GEKGEPGQGLRGLQGPPGKLGPPGNPGPSGSPGPKGQKGDPGKSPDGD 104

. : :*::.* *** . .* .* :**. : *

SP|P08254|MMP3_HUMAN PALSFDAVSTLRGEILIFKDRHFWRKSLRKLEPELHLISSFWPSLPSGVDAAYEVTSKDL 351

SP|P11226|MBL2_HUMAN SSLAASERKALQTEMA--------------------RI-KKWLTFSLGKQ---------- 133

:*: . .:*: *: * . * :: * :

SP|P08254|MMP3_HUMAN VFIFKGNQFWAIRGNEVRA--------------GYPRGIHTLGFPPTVRKIDA--AISDK 395

SP|P11226|MBL2_HUMAN ----VGNKFFLTNGEIMTFEKVKALCVKFQASVATPRNAAENGAIQNLIKEEAFLGITDE 189

**:*: .*: : . **. * .: * :* .*:*:

SP|P08254|MMP3_HUMAN EKNKTYFFV-----EDKYWRFDEKRNSMEPGFPKQIAEDFPGIDSKIDAVFEEFGFFYFF 450

SP|P11226|MBL2_HUMAN KTEGQFVDLTGNRLTYTNWNEGEPNN---------A------------------------ 216

:.: :. : . *. .* .*

SP|P08254|MMP3_HUMAN TGSSQLEFDPNAKKVTHTLKSNSWLNC-------------- 477

SP|P11226|MBL2_HUMAN ---------GSDEDCVLLLKNGQWNDVPCSTSHLAVCEFPI 248

. :. . **...* :

## Clarification of data analysis strategy

#### S-Fig 11: Data analysis workflow.

Visual representation of the steps of data analysis from raw data to z-scores as described in details in the Material and Method section.


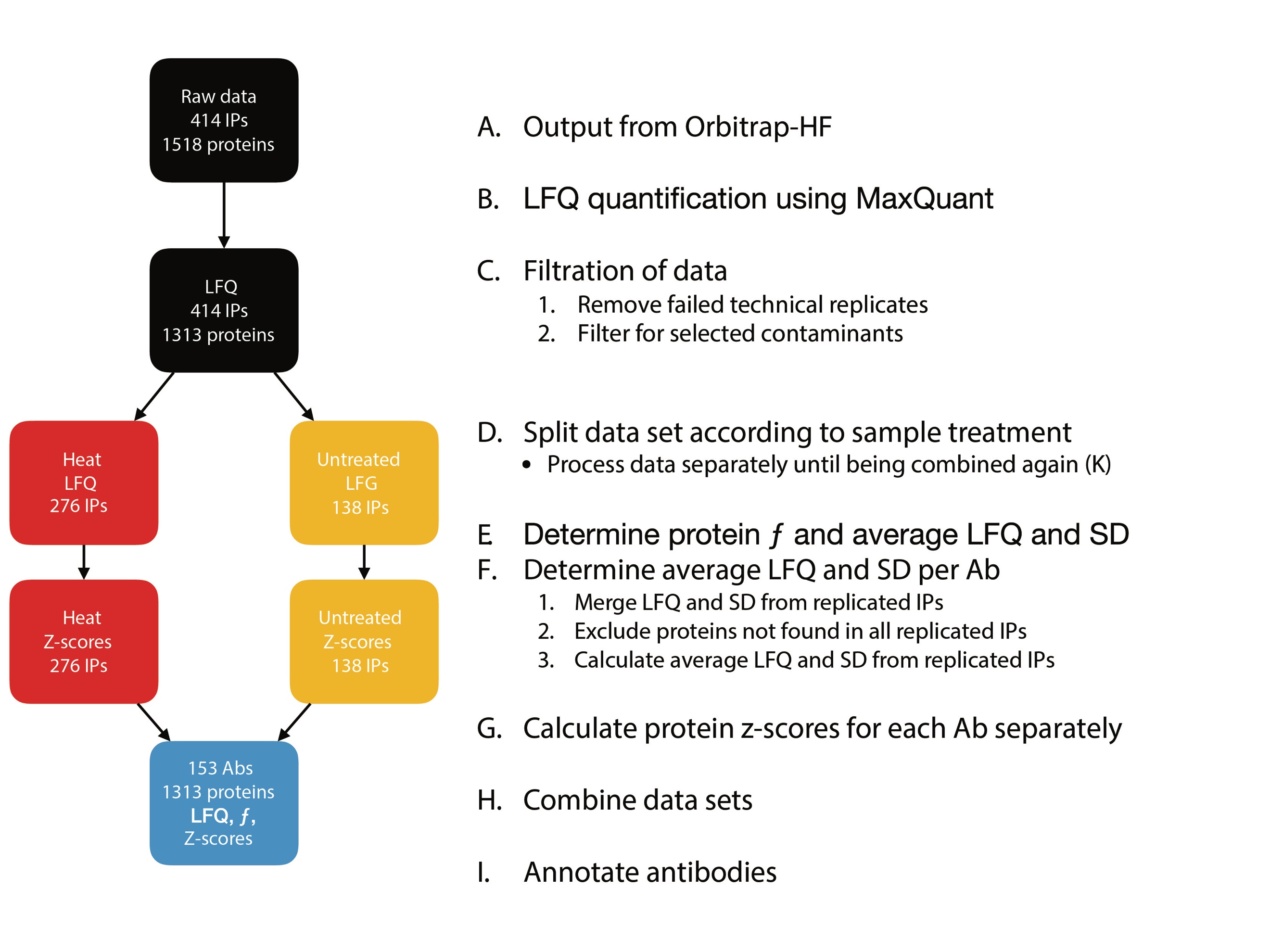


## Antibodies evaluation by protein arrays, WB and IP.

In plasma proteomics, current options for assessing the selectivity of antibodies can be offered by paired antibodies, protein arrays ^12^ or Western blot (WB) ^13^. Even if the three methods are each valuable in evaluating antibodies quality, validation of a previous discovery or qualification prior to a planned assay should be performed by a method resembling as much as possible the experimental conditions of the previous or intended application.

For both protein arrays and WB, the setup is that a surplus of antibodies is diluted in a solution and applied onto supports that present the antigens. Hence, there is generally minimal competition for binding sites between potential on- and off-targets as compared to antibodies being immobilized and applied to a complex solution. For an example given by anti-IL6R binder, we found application dependent recognition for five different antibodies. All five were classified as target-specific using protein arrays, however only three detected IL6R in plasma (**S**-**Excel Table**). It is consequently the composition of plasma, where 90% of protein content is assigned to 20 proteins, that poses a challenge for WB in terms of analytical resolution. In samples in which the concentrations of certain proteins dominate, the efficiency of how proteins migrate through the gel can be affected. This can influence the distribution of proteins in terms of their molecular mass and abundance; hence limits the amount of plasma loaded on the gel and so detectability of less abundant targets.

At first, we compared the classifications obtained here with existing scores of validation assigned by plasma WB to the same antibody’s Lot (HPA ID) within the Human Protein Atlas project ^13^. Noteworthy, the comparison is based on different samples and the samples chosen for WB analysis were depleted of albumin and IgG prior use ^14^. Nevertheless, we found that the assessment of 13 out of 104 antibodies (12%) provided supportive evidence by both methods. For antibodies raised against plasma proteins, the success rates for plasma IP (54%) was though higher than for plasma WB (32%) (**Supplementary Table 1**). When considering cellular proteins, the success rates were the same 33%. For WB however, uncertainty will remain unless other standards or comparative analyses are used to reference the detected bands. Until then, bands detected at the predicted molecular weight may still represent the recognition of an off-target molecule.

Consequently, IP provides an unequivocal identification of the target and could elucidate ambiguous WB results (see C1orf64, CEP162, E2F7, and CCL16 **S-Excel Table**, sheet: ”Antibodies_experim_annotation”). While WB may provide a more accessible technology for some labs, the information added by identification in MS will be required for an in-depth analysis of antibody selectivity. IP-MS can here be one option to enhance our understanding of binding to proteins, in particular for plasma.

#### **S-Table 1:** Comparison of validation rate on WB and IP-MS.

We used 104 antibodies previously assessed in plasma by WB and compared these to the IP-MS data. An antibody was considered passed by WB if scored 1,2 or 3, according to the Human Protein Atlas project.

|  | Plasma (MS)/Extracellular | Cellular | Sum |
| --- | --- | --- | --- |
| Method | N (%) | N (%) | N (%) |
| Pass by IP-MS + WB* | 11 (19) | 2 (4) | 13 (13) |
| Pass only by WB | 8 (13) | 13 (29) | 20(20) |
| Pass only by IP-MS | 21(35) | 13 (29) | 34 (33) |
| WB (Total) | 19 (32) | 15 (33 |  |
| IP-MS (Total) | 32 (54) | 15 (33) |  |
| Uncertain (IP-MS + WB*) | 19 (33) | 17 (38) | 36 (35) |
| Total* | 59 (100) | 45 (100) | 104* (100) |

*Only 104 antibodies were assessed both by IP and WB, and then compared.

# CONTENT OF SUPPLEMENTARY EXCEL TABLES

- “Selected targets”: Description of proteins for which antibodies were selected. (See **Supplementary References 23-108**)
- “Protein Annotation (GO)”: Protein annotation using GO terms.
- “Antibodies experim. annotations”: Antibodies used in the study, annotations about experimental conditions and data of validation by pIP and WB
- “Frequencies of identification”: List of proteins identified in pIP experiments and their frequencies of identification in heat treated or not heat treated plasma.
- “Batches Kruskal Wallis Test”: p-values for differences in intensity levels detected the between batches
- “Go enrichment analysis”: Differential GO analysis using TOPP cluster between frequent (> 20%) and less frequently occurring proteins (< 20%).
- “z-score >2.5”: List proteins for which a z-score > 2.5 was calculated containing targets, off-targets and potential interactors.
- “List of Peptides”: Peptides identified in pIP assays for the expected target proteins and overlapping with the antigen used to generate the antibodies.
- “Experimental Batches”: description of major differences in the experimental conditions for the 6 experimental batches analyzed.
- “Catalogs and Lot_numbers”: List of catalog and lot numbers for affinity reagents.
- “Antibodies against same protein”: proteins enriched by antibodies raised against the same target protein, using same or different protein fragments.

# SUPPLEMENTARY REFERENCES

1 Schwenk, J. M. *et al.* The Human Plasma Proteome Draft of 2017: Building on the Human Plasma PeptideAtlas from Mass Spectrometry and Complementary Assays. *J Proteome Res*, doi:10.1021/acs.jproteome.7b00467 (2017).

2 Uhlen, M. *et al.* Proteomics. Tissue-based map of the human proteome. *Science* **347**, 1260419, doi:10.1126/science.1260419 (2015).

3 Tamilselvi, E., Haripriya, D., Hemamalini, M., Pushpa, G. & Swapna, S. Association of disease severity with IL-1 levels in methotrexate-treated psoriasis patients. *Scand J Immunol* **78**, 545-553, doi:10.1111/sji.12117 (2013).

4 Borte, S. *et al.* Neonatal screening for severe primary immunodeficiency diseases using high-throughput triplex real-time PCR. *Blood* **119**, 2552-2555, doi:10.1182/blood-2011-08-371021 (2012).

5 Lilja, H., Ulmert, D. & Vickers, A. J. Prostate-specific antigen and prostate cancer: prediction, detection and monitoring. *Nat Rev Cancer* **8**, 268-278, doi:10.1038/nrc2351 (2008).

6 Stowe, R. P., Peek, M. K., Cutchin, M. P. & Goodwin, J. S. Plasma cytokine levels in a population-based study: relation to age and ethnicity. *J Gerontol A Biol Sci Med Sci* **65**, 429-433, doi:10.1093/gerona/glp198 (2010).

7 Del Valle-Pinero, A. Y. *et al.* Pro-inflammatory chemokine C-C motif ligand 16 (CCL-16) dysregulation in irritable bowel syndrome (IBS): a pilot study. *Neurogastroenterology and motility : the official journal of the European Gastrointestinal Motility Society* **23**, 1092-1097, doi:10.1111/j.1365-2982.2011.01792.x (2011).

8 Luc, G. *et al.* Plasma cystatin-C and development of coronary heart disease: The PRIME Study. *Atherosclerosis* **185**, 375-380, doi:10.1016/j.atherosclerosis.2005.06.017 (2006).

9 Murase, K. *et al.* Association between plasma neutrophil gelatinase associated lipocalin level and obstructive sleep apnea or nocturnal intermittent hypoxia. *PLoS One* **8**, e54184, doi:10.1371/journal.pone.0054184 (2013).

10 Nilsson, P. *et al.* Towards a human proteome atlas: high-throughput generation of mono-specific antibodies for tissue profiling. *Proteomics* **5**, 4327-4337 (2005).

11 Schwenk, J. M., Lindberg, J., Sundberg, M., Uhlen, M. & Nilsson, P. Determination of binding specificities in highly multiplexed bead-based assays for antibody proteomics. *Mol Cell Proteomics* **6**, 125-132, doi:10.1074/mcp.T600035-MCP200 (2007).

12 Sjöberg, R. *et al.* Exploration of high-density protein microarrays for antibody validation and autoimmunity profiling. *N Biotechnol* **33**, 582-592, doi:10.1016/j.nbt.2015.09.002 (2016).

13 Algenas, C. *et al.* Antibody performance in western blot applications is context-dependent. *Biotechnol J* **9**, 435-445, doi:10.1002/biot.201300341 (2014).

14 Eriksson, C., Schwenk, J. M., Sjoberg, A. & Hober, S. Affibody molecule-mediated depletion of HSA and IgG using different buffer compositions: a 15 min protocol for parallel processing of 1-48 samples. *Biotechnol Appl Biochem* **56**, 49-57, doi:10.1042/BA20100041 (2010).
